# Determining the Interaction in a Drug Combination using the Dose-based or Effect-based Method

**DOI:** 10.2174/011570159X347472250130111339

**Published:** 2025-02-06

**Authors:** Tinghe Yu, Tianran Yu, Xinya Li, Min Li

**Affiliations:** 1 Laboratory of Obstetrics and Gynecology, Second Affiliated Hospital, Chongqing Medical University, Chongqing, China;; 2 Chongqing Yangjiaping High School, Chongqing, China

**Keywords:** Drug combination, dose-effect, combination index, Chou’s method, Jin’s method, prodrug-enzyme therapy

## Abstract

The interaction in a drug combination can be assessed using either the Chou's or Jin’s method. The combination index in the former (*i.e*., CI-C) is calculated based on doses, while the latter (*i.e*., CI-J) is based on effects. This perspective demonstrates a consistency between 1/CI-C and CI-J when applied to both released and simulated data. Thus, 1/CI-C and CI-J are functionally equivalent for evaluating the drug interaction. Combining these two indices is preferred: a consistency shows a reliable verdict, and an inconsistency indicates a requirement for further analyses. However, it has been observed that evaluating released data raises certain concerns.

## 1 INTRODUCTION

Determination of the drug interaction is critical when developing a combination regimen. Addition and synergism are pharmacologically beneficial, but antagonism is undesirable; conversely, the opposite is appropriate for toxicological effects. The Chou’s method is widely used to evaluate the interaction.

f_a_/(1-f_a_) = (D/D_m_)^m^ Eq. (1)

f_a_ is the fraction affected (0<f_a_<1), D is the dose, D_m_ is the median-effect dose (*i.e*., half-maximal effective dose EC_50_), and m depicts the shape of a dose-effect curve. The interaction is assessed with the combination index (CI-C) based on actual (for the combination) and theoretical (for a drug alone) doses.

CI-C = (D_A|A+B_/D_A_)+(D_B|A+B_/D_B_) Eq. (2)

D_A|A+B_ and D_B|A+B_ are doses of A and B in the combination to produce a given effect, respectively. D_A_ or D_B_ is the dose required to cause that given effect when using A or B alone, which is calculated by Eq. 1; therefore, the dose-effect feature of each drug alone must be established before quantifying the interaction. A CI-C of <0.90, 0.90-1.10, or >1.10 represents synergism, addition, or antagonism, respectively [1-3]. Mathematically, these values correspond to 1/CI-C of >1.11, 0.91-1.11, and <0.91, respectively.

The method proposed by Jin is frequently used to determine the drug interaction, where the combination index (CI-J) is calculated using the effects observed.

CI-J = E_A+B_/(E_A_+E_B_-E_A_×E_B_) Eq. (3)

E_A_ or E_B_ is the effect induced by a drug alone, and E_A+B_ is an effect of the combination (doses of A and B in E_A+B_ are equal to those in E_A_ and E_B_, respectively). E_A_, E_B_, and E_A+B_ should be (0, 1). A CI-J of >1.15, 0.85-1.15, or <0.85 shows synergism, addition, or antagonism, respectively [4]. The advantages of Jin’s method are: i) acquiring the dose-effect feature of each drug alone is not mandatory, and ii) setting doses has more flexibility since doses are not directly used to calculate CI-J. Mathematically, 1/CI-C and CI-J are directly comparable.

In an *in vitro* cytotoxicity trial, the dead-cell fraction is f_a_ in Chou’s method or the effect in Jin’s method. Eq. 3 can be rewritten as:

CI-J = f_a_(A+B)/[f_a_(A)+f_a_(B)-f_a_(A)×f_a_(B)] Eq. (4)

These results indicate that cytotoxicity trials are a platform to determine the agreement between 1/CI-C and CI-J in assessing the drug interaction. It has been observed that these two indices may be compatible and comparable. Here, this hypothesis was tested to address the disagreement between these two assays.

**Fig. (1):**
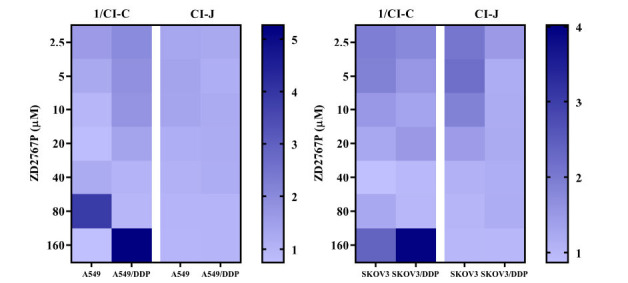
1/CI-C and CI-J in A549, A549/DDP (*left*), SKOV3, and SKOV3/DDP (*right*) cells. An agreement was observed in most cases; A549/DDP and SKOV3/DDP were cisplatin-resistant sublines (data were from Ref. [5, 6]). CI-C: combination index from the Chou’s method; CI-J: combination index from the Jin’s method.

This hypothesis was first tested on our previous data where ultrasound was used to modulate prodrug-enzyme therapy on cisplatin-sensitive and -resistant cancer cells [5, 6]. Of those 28 data pairs in 4 cell lines, 1/CI-C agreed with CI-J in 25 (89.3%) pairs (*i.e*., a beneficial interaction). For the other 3 pairs, 1/CI-C indicated an undesirable interaction, but CI-J showed a beneficial one (Fig. **1**) [5, 6]. When summarizing data in each cell line, both 1/CI-C and CI-J showed a beneficial interaction; therefore, those 3 disagreements may be due to experimental errors or heterogeneity.

Reevaluating available data is an efficient verification strategy [7]. This hypothesis was tested on drug data released in 2021-2023. The agreement rate between listed and recalculated 1/CI-C was 86.2% (κ=0.64, 95% confidence interval: 0.54-0.74; *p <* 0.0001), demonstrating the reliability of the reevaluations. 1/CI-C agreed with CI-J in 81.4% items (κ=0.52, 95% confidence interval: 0.41-0.63; *p <* 0.0001). When adopting recalculated 1/CI-C, the agreement rate between 1/CI-C and CI-J was 90.3% (κ=0.74, 95% confidence interval: 0.65-0.83; *p <* 0.0001) (Table **S1**, Fig. **S1**). These findings manifested that 1/CI-C and CI-J were functionally equivalent.

Simulations were performed (D_m_(A)=10, D_m_(B)=2-20, D_m_(A+B)=2-20, and m=2, 0.5, 1; with 6 doses). Agreement rates between 1/CI-C and CI-J were 73.7% (κ=0.48, 95% confidence interval: 0.42-0.54; *p <* 0.0001), 79.8% (κ=0.59, 95% confidence interval: 0.53-0.65; *p <* 0.0001), and 91.8% (κ=0.84, 95% confidence interval: 0.79-0.88; *p <* 0.0001) at m=2, 0.5, or 1, respectively. Therefore, m impacted the agreement rate. More inconsistencies were noted when D_m_(A+B) was near to D_m_(B) (Tables **S2-S4**, Fig. **S2**). A smaller difference between D_m_(A+B) and D_m_(B) indicated a reduced shift in the dose-effect curve of the combination relative to that of a drug alone, thereby increasing the risk of misinterpreting interactions. The disagreement between 1/CI-C and CI-J serves as an indication for careful verification.

## 2 CERTAIN CONCERNS

Evaluating released drug data raises certain issues that require specific considerations in designing/performing a combination therapy and assessing the interaction. The following aspects are important in evaluating data.

### 2.1 EC_50_

Setting doses in the combination based on EC_50_ of each drug is the most effective approach [3]. Further, EC_50_ is frequently adopted to elucidate underlying mechanisms in preclinical trials. The four-parameter dose-effect model was used to calculate EC_50_ in certain trials [8-10]. This method may lead to errors since the dead-cell fractions are relative values. The four-parameter model approximately considers the maximum fractions as the top and the minimal fractions as the bottom, but a top of ≈1 with a bottom of ≈0 is rare. Indeed, EC_50_ from the four-parameter analysis is the relative EC_50_ (*i.e*., cannot be directly compared) and is commonly less than the actual EC_50_ (*e.g*., the four-parameter function indicated that EC_50_ of escin were 2.24, 3.27 and 3.39 μM for HepG2, PLC/PRF5 and Huh7 cells, respectively; however, EC_50_ were 38.1, 38.6 and 22.3 μM when using the Chou’s method, respectively) [8]. Extremely, the maximal dead-cell fraction is <0.5, *i.e*., the actual EC_50_ can only be extrapolated; however, the four-parameter analysis will give a plausible value [9]. Adopting such EC_50_ led to illogical results: dead-cell fractions were ≤0, or values in the combination were inconsistent with those in the dose-effect curve of a drug alone (*e.g*., in the trial by Falcão *et al*., the highest dead-cell fraction of 0.1-100 μM 5-fluorouracil alone was ≈0.3 in HT-29 cells, but 3.78 μM 5-fluorouracil alone led to a dead-cell fraction of 0.5 in the combination therapy), thereby limiting or distorting the interaction evaluation [9, 10]. As per our experience, the Chou’s method is reliable in determining EC_50_, and the dose range should be rationally set.

### 2.2 Dose-effect Curve

D_A_ and D_B_ in Eq. 2 are calculated using the dose-effect curve of each drug alone. In the trial by Falcão *et al*., all dead-cell fractions of green propolis were <0, *i.e*., the dose-effect property could not be summarized. There were only 2 logical data points for red or brown propolis, which inaccurately outlined the dose-effect feature and eventually led to biases in D_A_ (Table **S1**) [10]. The dose-effect feature of a drug alone could be comprehensively understood with ≥5 rational data points (0<f_a_<1). The verdict needs particular concerns when both D_m_(A) and D_m_(B) are extrapolated values (*i.e*., beyond the set doses). The Jin’s method is preferred in such cases.

### 2.3 Harmonization of the Chou’s and Jin’s Methods

The Chou’s method is based on doses, and the Jin’s method utilizes effects. Here the findings of 1/CI-C and CI-J were consistent in most cases. This can be explained from the perspective of pharmacokinetics-pharmacodynamics: the peak concentration and the area under the concentration *vs.* time curve of A are altered by B, ultimately modulating the effect of A and its contribution to the overall effect of the combination [11-13]. Therefore, the results obtained from the Chou’s method can be verified by the Jin’s method and vice versa (comparisons are listed in Table **S5**). An inconsistency needs further evaluations.

### 2.4 Data Interpretation

The interaction should be determined using overall 1/CI-C and CI-J. The threshold effect can only be confirmed when all interactions shift in the opposite direction after a specific dose. A change at several medium doses may be a fluctuation due to experimental errors. A reversal at very high or low doses (*e.g*., antagonism at the lowest doses) requires further trials at several higher/lower doses, or other assays.

For *in vivo* therapy, setting multiple doses to acquire the dose-effect feature of a drug alone is impossible sometimes, limiting applications of the Chou’s method. The Jin’s method can operate, whereas other assays, such as pharmacokinetics, can serve as an adjunct for further validation. Using artificial intelligence to evaluate the drug interaction is in development, particularly for bulk data [14, 15]. Chou’s and Jin’s methods are used for routine analysis of small data, or as a screening or validation tool.

## CONCLUSION

In summary, 1/CI-C and CI-J for evaluation of the drug interaction are functionally equivalent. 1/CI-C and CI-J can be directly compared, and these two assays are complementary. Combining these two indices should be encouraged: a consistency indicates a reliable verdict, and an inconsistency shows that further evaluations are needed.

## LIST OF ABBREVIATIONS

CI-C: Combination Index from the Chou's Method
CI-J: Combination Index from the Jin's Method
EC_50_: Half-maximal Effective Dose
