# Supplementary material for: Determining the Interaction in a Drug Combination using the Dose-based or Effect-based Method
Source: Curr Neuropharmacol. 2025 Feb 6;23(10):1260–3. doi: 10.2174/011570159X347472250130111339 (PMC12307990; doi:10.2174/011570159X347472250130111339)
Supplement: Supplementary file 1 [file CN-23-10-1260_SD1.pdf]

Supplementary Material

Determining the Interaction in a Drug Combination using the Dose-based or Effect-based Method

Tinghe Yu[1,*], Tianran Yu[2], Xinya Li[1] and Min Li[1,*]

[1]Laboratory of Obstetrics and Gynecology, Second Affiliated Hospital, Chongqing Medical University, Chongqing, China; [2]Chongqing Yangjiaping High School, Chongqing, China

1875-6190/25 $65.00+.00 © 2025 Bentham Science Publishers

Table S1. List of 1/CI-C (Chou's method) and CI-J (Jin's method) on released data.

Trial	Cell	Drug		1/CI (Chou)		Reevaluation				Notes
						1/CI (Chou)		CI (Jin)		
Environ Toxicol 2024,39,840		Sorafenib (μM)	Escin (μM)							
	HepG2	0.1	3	0.81	–	0.34	–	0.51	–	
		0.3		0.74	–	0.40	–	0.55	–	
		1		0.51	–	0.45	–	0.61	–	
		3		1.59	+	1.82	+	0.96	+	
		10		1.41	+	2.16	+	0.97	+	
		30		3.12	+	1.90	+	0.98	+	
		100		0.75	–	2.94	+	1.01	+	
		0.1	10	0.43	–	0.40	–	0.56	–	
		0.3		0.96	+	0.59	–	0.62	–	
		1		1.25	+	0.85	–	0.71	–	
		3		1.43	+	2.72	+	0.94	+	
		10		1.23	+	3.34	+	0.96	+	
		30		3.45	+	9.71	+	1.07	+	
		100		3.57	+	4.61	+	1.01	+	
		0.1	30	0.33	–	0.36	–	0.66	–	
		0.3		0.46	–	0.47	–	0.70	–	
		1		0.45	–	0.56	–	0.72	–	
		3		1.61	+	2.18	+	0.92	+	
		10		2.27	+	6.71	+	1.03	+	
		30		8.33	+	4.74	+	1.00	+	
		100		2.70	+	2.92	+	0.98	+	
	PLC/PRF5	0.1	3	0.14	–	0.07	–	0.32	–	
		0.3		0.47	–	0.24	–	0.47	–	
		1		0.62	–	0.51	–	0.61	–	
		3		1.64	+	1.63	+	0.89	+	
		10		1.47	+	1.46	+	0.98	+	
		30		0.89	–	0.88	–	0.95	+	

		100		1.85	+	1.52	+	0.99	+	
		0.1	10	0.68	–	0.11	–	0.39	–	
		0.3		0.99	+	0.28	–	0.52	–	
		1		0.99	+	0.49	–	0.61	–	
		3		1.69	+	1.85	+	0.86	+	
		10		1.23	+	2.07	+	0.98	+	
		30		1.11	+	1.32	+	0.97	+	
		100		2.44	+	2.55	+	1.00	+	
		0.1	30	0.54	–	0.29	–	0.67	–	
		0.3		0.86	–	0.60	–	0.78	–	
		1		0.98	+	0.63	–	0.76	–	
		3		2.04	+	2.90	+	0.97	+	
		10		1.75	+	5.71	+	1.09	+	
		30		1.45	+	2.54	+	1.02	+	
		100		1.20	+	14.49	+	1.03	+	
	Huh-7	0.1	3	0.31		0.17				Cell-death percentages at 0.1 and 0.3 μM sorafenib were unavailable.
		0.3		0.69		0.49				
		1		1.25	+	0.90	–	0.62	–	
		3		2.56	+	2.26	+	0.97	+	
		10		2.44	+	3.17	+	1.07	+	
		30		1.67	+	2.78	+	1.03	+	
		100		0.97	+	1.87	+	1.05	+	
		0.1	10	0.01		0.25				Cell-death percentages at 0.1 and 0.3 μM sorafenib were unavailable.
		0.3		0.15		0.39				
		1		0.67	–	0.69	–	0.65	–	
		3		2.08	+	1.65	+	0.89	+	
		10		1.89	+	4.27	+	1.08	+	
		30		1.72	+	3.18	+	1.01	+	
		100		0.98	+	2.18	+	1.03	+	
		0.1	30	0.17		0.15				Cell-death percentages at 0.1 and 0.3 μM sorafenib were unavailable.
		0.3		0.45		0.18				

		1		1.14	+	0.65	–	0.77	–	
		3		3.12	+	2.08	+	1.01	+	
		10		2.94	+	3.26	+	1.01	+	
		30		2.17	+	5.43	+	1.03	+	
		100		0.52	–	3.16	+	1.02	+	
Exp Hematol Oncol 2023,12,23		Chidamide (µM)	Cladribine (µM)							
	U937	0.5	0.01	1.18	+	1.33	+	1.16	+	The cell-death percentage at 16 µM chidamide was unavailable.
		1		1.09	+	1.29	+	1.11	+	
		2		1.18	+	1.62	+	1.26	+	
		4		0.94	+	1.59	+	1.21	+	
		8		0.84	–	1.71	+	1.05	+	
		16		2.03		5.62				
		0.5	0.02	1.67	+	1.82	+	1.38	+	The cell-death percentage at 16 µM chidamide was unavailable.
		1		1.63	+	1.87	+	1.31	+	
		2		1.43	+	1.80	+	1.25	+	
		4		1.15	+	2.69	+	1.32	+	
		8		0.79	–	1.41	+	0.98	+	
		16		1.49		3.55				
	THP-1	0.3	0.02	0.76		0.59				The cell-death percentage at 0.3 µM chidamide was 0.
		0.5		1.10	+	0.91	+	1.79	+	
		1		1.57	+	1.35	+	3.02	+	
		2		2.01	+	1.92	+	3.16	+	
		4		3.06	+	3.17	+	2.60	+	
		8		3.72	+	4.46	+	1.70	+	
		0.3	0.04	0.96		0.82				The cell-death percentage at 0.3 µM chidamide was 0.
		0.5		1.19	+	1.06	+	1.59	+	
		1		1.55	+	1.51	+	2.01	+	
		2		2.00	+	2.04	+	2.07	+	
		4		2.88	+	3.53	+	1.89	+	
		8		3.33	+	3.83	+	1.44	+	

	MV4-11	0.1	0.01	1.42						The cell-death percentage at 0.01 µM cladribine was unavailable.
		0.2		1.13						
		0.4		1.02						
		0.8		0.90						
		1.6		1.73						
		3.2		0.93						
		0.1	0.02	1.47	+	1.64	+	1.34	+	The cell-death percentage at 3.2 µM chidamide was unavailable.
		0.2		1.37	+	1.57	+	1.35	+	
		0.4		1.36	+	1.78	+	1.22	+	
		0.8		1.22	+	1.77	+	1.13	+	
		1.6		1.12	+	2.07	+	1.04	+	
		3.2		2.04						
Anticancer Agents Med Chem 2023,23,779		Telaglenastat (µM)	Epigallocatechin-3-gallate (µM)							
	KM3/BTZ	1.25	40	0.94	+	1.15	+	0.72	–	
		2.5		1.31	+	1.61	+	1.06	+	
		5		1.09	+	1.36	+	1.00	+	
		1.25	80	1.32	+	1.57	+	0.96	+	
		2.5		1.96	+	2.31	+	1.34	+	
		5		5.75	+	6.58	+	1.68	+	
		1.25	120	3.49	+	3.89	+	1.46	+	
		2.5		3.88	+	4.35	+	1.67	+	
		5		6.19	+	6.90	+	1.68	+	
Mole-cules 2023,28, 3393		Green propolis (µg/ml)	5-fluorouracil (µM)							
	HT-29	6.25	3.78	2.04						Cell-death percentages of green propolis were <0.
		12.5		0.81						
		25		<0.01						
		50		0.58						
		100		16.67						

		Red propolis (μg/ml)								
		6.25	3.78	2.13		0.34				Cell-death percentages at 6.25-25 μg/ml red propolis were <0.
		12.5		1.39		0.12				
		25		0.84		0.05				
		50		1.05	+	0.45	–	0.23	–	
		100		1.52	+	1.70	+	1.06	+	
		Brown propolis (μg/ml)								
		6.25	3.78	2.70		0.60				Cell-death percentages at 6.25-25 μg/ml brown propolis were <0.
		12.5		2.38		0.86				
		25		1.09		0.12				
		50		0.68	–	0.08	–	0.12	–	
		100		1.56	+	1.59	+	1.11	+	
		Green propolis (μg/ml)	Fluphenazine (μM)							
		6.25	1.86	<0.01						Cell-death percentages of green propolis, and at 6.25 μg/ml green propolis + 1.86 μM fluphenazine were <0.
		12.5		<0.01						
		25		0.02						
		50		0.13						
		100		2.78						
		Red propolis (μg/ml)								
		6.25	1.86	<0.01						Cell-death percentages at 6.25-25 μg/ml red propolis, and at 6.25-25 μg/ml red propolis + 1.86 μM fluphenazine were <0.
		12.5		<0.01						
		25		<0.01						
		50		0.21	–	0.21	–	0.43	–	
		100		1.37	+	1.57	+	1.07	+	
		Brown propolis (μg/ml)								
		6.25	1.86	<0.01		<0.01				Cell-death percentages at 6.25-25 μg/ml brown propolis were <0.
		12.5		<0.01		0.01				
		25		0.05		0.06				

		50		0.19	–	0.20	–	0.46	–	
		100		0.95	+	0.95	+	0.98	+	
Pharma-ceutics 2022,14,511		Honeybee venom (µg/ml)	5-fluorouracil (µM)							
	HT-29	6.25	3.56	2.38		0.28				Cell-death percentages at 6.25 and 12.5 µg/ml honeybee venom were ≤0.
		12.5		2.94		1.01				
		25		2.13	+	1.43	+	1.09	+	
		50		1.30	+	1.71	+	0.98	+	
		100		0.65	–	0.86	–	0.98	+	
			Fluphenazine (µM)							
		6.25	1.86	<0.01		0.01				Cell-death percentages at 6.25 and 12.5 µg/ml honeybee venom were ≤0.
		12.5		0.23		0.24				
		25		1.72	+	1.46	+	1.09	+	
		50		1.20	+	1.43	+	0.95	+	
		100		0.62	–	0.75	–	0.95	+	
			Fluoxetine (µM)							
		6.25	6.12	5.00		8.77				Cell-death percentages at 6.25 and 12.5 µg/ml honeybee venom were ≤0.
		12.5		3.70		6.99				
		25		2.27	+	4.31	+	1.24	+	
		50		1.27	+	2.44	+	1.00	+	
		100		0.68	–	1.31	+	1.00	+	
			Sertraline (µM)							
		6.25	2.45	6.67		9.09				Cell-death percentages at 6.25 and 12.5 µg/ml honeybee venom, and at 2.45 sertraline were ≤0.
		12.5		4.76		9.43				
		25		2.63		5.13				
		50		1.39		2.68				
		100		0.71		1.37				
			Thioridazine (µM)							
		6.25	4.26	7.69		9.80				Cell-death percentages at 6.25 and 12.5 µg/ml honeybee venom were ≤0.
		12.5		4.76		7.09				

		25		2.56	+	4.15	+	1.17	+	
		50		1.33	+	2.27	+	0.98	+	
		100		0.69	–	1.19	+	0.98	+	
			Doxorubicin (μM)							
	MCF-7	6.25	0.17	0.03		<0.01				The cell-death percentage at 6.25 μg/ml honeybee venom was <0.
		12.5		0.59	–	0.09	–	0.52	–	
		25		1.59	+	1.46	+	0.85	+	
		50		0.88	–	0.91	+	0.87	+	
		100		0.47	–	0.39	–	0.84	–	
			Fluphenazine (μM)							
		6.25	2.68	1.56		0.49				Cell-death percentages at 6.25 μg/ml honeybee venom, and at 2.68 μM fluphenazine were <0.
		12.5		1.82		0.88				
		25		1.49		1.18				
		50		0.84		0.66				
		100		0.45		0.35				
			Fluoxetine (μM)							
		6.25	7.78	2.94		2.94				Cell-death percentages at 6.25 μg/ml honeybee venom, and at 7.78 μM fluoxetine were <0.
		12.5		2.13		2.17				
		25		1.39		1.43				
		50		0.83		0.85				
		100		0.46		0.52				
			Sertraline (μM)							
		6.25	2.22	3.12		1.95				Cell-death percentages at 6.25 μg/ml honeybee venom, and at 2.22 μM sertraline were <0.
		12.5		3.03		3.66				
		25		1.89		2.97				
		50		0.99		1.66				
		100		0.52		0.89				
			Thioridazine (μM)							
		6.25	5.72	3.70		3.95				The cell-death percentage at 6.25 μg/ml honeybee venom was <0.
		12.5		2.63	+	3.29	+	1.18	+	
		25		1.64	+	2.29	+	0.94	+	

		50		0.94	+	1.43	+	0.94	+	
		100		0.51	–	0.81	–	0.94	+	
Phar-mazie 2022,77,59		Afatinib (µM)	Enzalutamide (µM)							
	22RV1	0.03	1	9.26	+	4.76	+	3.53	+	
		0.1	3	6.17	+	3.38	+	1.99	+	
		0.3	10	5.29	+	3.42	+	3.02	+	
		1	30	2.27	+	1.45	+	1.02	+	
		3	100	1.79	+	1.53	+	0.94	+	
		Gefitinib (µM)								
		1	1	4.61		2.48				The cell-death percentage at 1 µM gefitinib was <0.
		3	3	1.91	+	1.08	+	0.94	+	
		10	10	1.55	+	1.31	+	1.72	+	
		30	30	1.05	+	1.63	+	0.96	+	
		100	100	0.62	–	2.47	+	0.99	+	
		Erlotinib (µM)								
		0.3	1	0.53						Cell-death percentages at 0.3 and 1 µM erlotinib, at 1 µM enzalutamide, and at 0.3 µM erlotinib + 1 µM enzalutamide were ≤0.
		1	3	2.36		0.83				
		3	10	1.67	+	1.38	+	2.46	+	
		10	30	0.90	–	1.09	+	1.02	+	
		30	100	0.37	–	0.61	–	0.88	+	
		Sorafenib (µM)								
		0.1	1	0.99		0.48				The cell-death percentage at 0.1 µM sorafenib was <0.
		0.3	3	2.20	+	1.26	+	1.39	+	
		1	10	1.12	+	0.82	–	1.20	+	
		3	30	0.94	+	1.01	+	0.97	+	
		10	100	0.53	–	0.77	–	0.93	+	
		Dacomitinib (µM)								
		0.1	1	3.10		2.20				The cell-death percentage at 0.1 µM dacomitinib was <0.
		0.3	3	1.32	+	0.95	+	0.86	+	

		1	10	1.24	+	1.06	+	1.33	+	
		3	30	0.97	+	1.10	+	0.93	+	
		10	100	0.67	–	1.29	+	0.97	+	
		Afatinib (µM)								
	C4-2	0.1	1	1.12	+	0.92	+	0.87	+	
		0.3	3	0.77	–	0.48	–	0.65	–	
		1	10	0.38	–	0.42	–	0.62	–	
		3	30	0.20	–	0.20	–	0.64	–	
		10	100	3.30	+	3.70	+	0.99	+	
		Gefitinib (µM)								
		1	0.3	8.55		9.52				Cell-death percentages at 1 and 3 µM gefitinib were ≤0.
		3	1	5.26		7.30				
		10	3	2.69	+	3.12	+	0.96	+	
		30	10	1.80	+	3.83	+	0.94	+	
		100	30	0.70	–	2.22	+	0.82	–	
		Erlotinib (µM)								
		1	0.3	0.23		0.34				The cell-death percentage at 0.3 µM enzalutamide was <0.
		3	1	0.60	–	0.96	+	0.72	–	
		10	3	3.60	+	10.99	+	0.85	+	
		30	10	1.19	+	6.49	+	0.85	+	
		100	30	0.42	–	4.20	+	0.79	–	
		Sorafenib (µM)								
		0.1	1	2.33	+	2.47	+	1.06	+	
		0.3	3	0.85	–	0.82	–	0.70	–	
		1	10	0.78	–	0.79	–	0.76	–	
		3	30	0.56	–	0.50	–	0.80	–	
		10	100	5.43	+	4.93	+	0.97	+	
		Dacomitinib (µM)								
		0.1	1	6.13	+	4.46	+	0.99	+	
		0.3	3	1.23	+	1.49	+	0.72	–	
		1	10	3.14	+	1.36	+	0.68	–	

		3	30	0.19	–	0.19	–	0.55	–	
		10	100	0.62	–	1.06	+	0.83	–	
		Afatinib (μM)								
	DU145	0.03	1	4.31	+	4.15	+	1.14	+	
		0.1	3	3.88	+	3.47	+	1.41	+	
		0.3	10	2.15	+	1.87	+	0.99	+	
		1	30	1.10	+	1.03	+	0.97	+	
		3	100	1.98	+	2.25	+	0.97	+	
		Gefitinib (μM)								
		1	1	1.07		1.45				The cell-death percentage at 1 μM enzalutamide was <0.
		3	3	1.87	+	2.16	+	1.41	+	
		10	10	1.60	+	1.88	+	1.11	+	
		30	30	0.84	–	1.18	+	0.84	–	
		100	100	0.42	–	0.68	–	0.83	–	
		Erlotinib (μM)								
		1	1	5.15	+	4.81	+	1.41	+	
		3	3	9.43	+	9.43	+	1.52	+	
		10	10	4.88	+	5.71	+	0.99	+	
		30	30	3.14	+	3.83	+	1.06	+	
		100	100	1.92	+	2.64	+	1.06	+	
		Sorafenib (μM)								
		0.3	1	9.71	+	10.53	+	3.91	+	
		1	3	4.33	+	4.67	+	1.19	+	
		3	10	1.35	+	1.63	+	0.92	+	
		10	30	1.02	+	1.07	+	1.14	+	
		30	100	2.85	+	4.10	+	1.12	+	
		Dacomitinib (μM)								
		0.3	1	2.86	+	3.53	+	1.06	+	
		1	3	0.71	–	0.76	–	0.78	–	
		3	10	0.49	–	0.55	–	0.82	–	
		10	30	0.75	–	0.66	–	0.92	+	

		30	100	35.71	+	>100	+	1.01	+	
		Afatinib (µM)								
	LNCaP	0.3	1	1.49	+	1.77	+	0.79	–	
		1	3	1.34	+	1.41	+	0.77	–	
		3	10	0.62	–	0.76	–	0.80	–	
		10	30	1.53	+	1.74	+	0.87	+	
		30	100	1.31	+	2.12	+	0.89	+	
		Gefitinib (µM)								
		1	0.3	1.18		0.93				The cell-death percentage at 1 µM gefitinib was <0
		3	1	0.75	–	0.66	–	0.84	–	
		10	3	1.66	+	1.68	+	0.94	+	
		30	10	0.69	–	0.78	–	0.84	–	
		100	30	0.39	–	0.51	–	0.89	+	
		Erlotinib (µM)								
		1	0.3	9.26		0.01				The cell-death percentage at 1 µM erlotinib was <0
		3	1	6.17	+	0.18	–	0.48	–	
		10	3	5.29	+	0.20	–	0.61	–	
		30	10	2.27	+	2.03	+	0.97	+	
		100	30	1.79	+	2.02	+	0.94	+	
		Sorafenib (µM)								
		0.1	1	1.43	+	0.55	–	0.59	–	
		0.3	3	1.43	+	0.83	–	0.75	–	
		1	10	0.59	–	0.48	–	0.81	–	
		3	30	1.63	+	0.76	–	0.90	+	
		10	100	1.26	+	2.44	+	0.98	+	
		Dacomitinib (µM)								
		0.1	1	1.29	+	1.36	+	0.79	–	
		0.3	3	0.70	–	0.78	–	0.78	–	
		1	10	0.41	–	0.40	–	0.81	–	
		3	30	2.89	+	3.16	+	0.99	+	
		10	100	0.76	–	1.81	+	0.99	+	

		Afatinib (µM)								
	PC3	0.03	1	2.62		2.71				The cell-death percentage at 1 µM enzalutamide was <0.
		0.1	3	1.04	+	0.99	+	1.06	+	
		0.3	10	0.42	–	0.49	–	1.02	+	
		1	30	0.37	–	0.43	–	1.00	+	
		3	100	1.39	+	8.47	+	1.00	+	
		Gefitinib (µM)								
		0.3	1	0.75		0.62				The cell-death percentage at 1 µM enzalutamide was <0.
		1	3	0.81	–	0.65	–	0.68	–	
		3	10	1.17	+	1.39	+	1.36	+	
		10	30	0.39	–	0.42	–	0.76	–	
		30	100	0.68	–	2.73	+	0.89	+	
		Erlotinib (µM)								
		0.3	1	0.78		0.61				The cell-death percentage at 1 µM enzalutamide was <0.
		1	3	1.13	+	1.11	+	1.02	+	
		3	10	0.92	+	1.44	+	0.84	–	
		10	30	0.53	–	0.92	+	0.91	+	
		30	100	0.64	–	2.67	+	1.02	+	
		Sorafenib (µM)								
		0.1	1	0.68						Cell-death percentages at 0.1 and 0.3 µM sorafenib, at 1 µM enzalutamide, and at 0.1 µM sorafenib + 1 µM enzalutamide were <0.
		0.3	3	3.69		1.75				
		1	10	1.82	+	1.63	+	2.19	+	
		3	30	0.89	–	1.18	+	1.06	+	
		10	100	0.44	–	0.94	+	0.96	+	
		Dacomitinib (µM)								
		0.1	1	1.95		1.90				The cell-death percentage at 1 µM enzalutamide was <0.
		0.3	3	0.96	+	0.94	+	1.10	+	
		1	10	0.62	–	0.62	–	0.96	+	
		3	30	0.85	–	1.35	+	1.00	+	
		10	100	22.73	+	1.05	+	1.00	+	

Phytomedicine 2021,86,153553		5-fluorouracil (μM)	Tenacissoside G (μM)							
	RKO	0.5	25	1.37	+	1.44	+	1.15	+	
		1		1.49	+	1.52	+	1.15	+	
		2		1.59	+	1.67	+	1.30	+	
		4		1.59	+	1.64	+	1.22	+	
		8		1.37	+	1.43	+	1.01	+	
		0.5	50	1.52	+	1.47	+	1.21	+	
		1		1.69	+	1.57	+	1.20	+	
		2		1.85	+	1.69	+	1.26	+	
		4		2.27	+	1.83	+	1.23	+	
		8		3.57	+	2.71	+	1.27	+	
		0.5	100	1.06	+	0.96	+	0.85	+	
		1		1.30	+	1.14	+	0.90	+	
		2		1.49	+	1.31	+	0.97	+	
		4		1.92	+	1.63	+	1.03	+	
		8		1.89	+	1.68	+	0.98	+	
	LoVo	0.5	25	1.20	+	1.23	+	1.10	+	
		1		1.27	+	1.37	+	1.15	+	
		2		1.20	+	1.23	+	1.15	+	
		4		1.52	+	1.54	+	1.24	+	
		8		1.64	+	1.73	+	1.13	+	
		0.5	50	1.33	+	1.35	+	1.16	+	
		1		1.45	+	1.47	+	1.18	+	
		2		1.54	+	1.53	+	1.21	+	
		4		1.79	+	1.78	+	1.23	+	
		8		4.35	+	4.12	+	1.45	+	
		0.5	100	1.37	+	1.33	+	1.05	+	
		1		1.47	+	1.41	+	1.05	+	
		2		1.59	+	1.54	+	1.09	+	

		4		1.64	+	1.62	+	1.07	+	
		8		1.89	+	1.82	+	1.04	+	
Biomedicines 2021,9,1190		Cisplatin (µM)	Mitotane (µM)							
	NCI-H295R	3.125	25	0.25						Cell-death percentages 6.25-25 µM cisplatin, at 25 µM mitotane, and at 3.125 µM cisplatin + 25 µM mitotane were <0.
		6.25		1.38		1.98				
		12.5		3.97		2.54				
		25		2.03		1.52				
		Doxorubicin (µM)								
		0.15625	25	21.57		8.13				The cell-death percentage at 25 µM mitotane was <0.
		0.3125		22.45		8.33				
		0.625		26.75		9.01				
		1.25		23.36		8.47				
		Etoposide (µM)								
		4.6875	25	1.46		1.12				The cell-death percentage at 25 µM mitotane was <0.
		9.375		20.77		8.40				
		18.75		61.39		13.33				
		37.5		69.49		14.29				
		Doxorubicin (µM)	Etoposide (µM)							
		0.15625	4.6875	1.04	+	<0.01	–	0.45	–	
		0.3125	9.375	0.91	+	0.01	–	0.54	–	
		0.625	18.75	1.91	+	0.16	–	0.70	–	
		1.25	37.5	13.57	+	33.33	+	0.94	+	
		Cisplatin (µM)	Etoposide (µM)							
		3.125	4.6875	0.09	–	<0.01	–	0.18	–	Cell-death percentages at 6.25-25 µM cisplatin were <0.
		6.25	9.375	0.03		<0.01				
		12.5	18.75	0.72		0.02				
		25	37.5	3.26		1.97				
		Cisplatin (µM)	Doxorubicin (µM)							

		3.125	0.15625	<0.01						Cell-death percentages at 6.25-25 µM cisplatin, and at 3.125/6.25 µM cisplatin + 0.15625/0.3125 µM doxorubicin were <0.
		6.25	0.3125	<0.01						
		12.5	0.625	0.24		<0.01				
		25	1.25	1.16		2.90				
		Cisplatin (µM)	Mitotane (µM)							
	SW13	1.5625	50			1.67				Cell-death percentages at 1.5625 and 3.125 µM cisplatin were <0.
		3.125		0.25		2.56				
		6.25		1.38	+	2.14	+	3.63	+	
		12.5		3.97	+	1.70	+	2.08	+	
		25		2.03	+	1.02	+	1.02	+	
		Doxorubicin (µM)								
		0.0781	50							
		0.15625		0.08						Cell-death percentages at 0.0781-0.625 µM doxorubicin, and at 0.15625-0.625 µM doxorubicin + 50 µM mitotane were ≤0.
		0.3125		0.08						
		0.625		0.07						
		1.25		2.17	+			0.97	+	
		Etoposide (µM)								
		4.6875	50	0.12	–	0.32	–	0.72	–	
		9.375		0.16	–	0.24	–	0.47	–	
		18.75		0.42	–	0.44	–	0.72	–	
		37.5		1.44	+	1.20	+	1.11	+	
		Doxorubicin (µM)	Etoposide (µM)							
		0.15625	4.6875	15.52		15.62				Cell-death percentages at 0.15625-0.625 µM doxorubicin were <0.
		0.3125	9.375	12.58		18.52				
		0.625	18.75	23.42		83.33				
		1.25	37.5	17.53	+	28.57	+	1.20	+	
		Cisplatin (µM)	Etoposide (µM)							
		3.125	4.6875	0.55		0.34				The cell-death percentage at 3.125 µM cisplatin was <0.
		6.25	9.375	0.16	–	0.11	–	0.14	–	
		12.5	18.75	0.21	–	0.13	–	0.18	–	
		25	37.5	0.47	–	0.39	–	0.51	–	

		Cisplatin (μM)	Doxorubicin (μM)							
		3.125	0.15625	1.60		9.09				Cell-death percentages at 3.125 μM cisplatin, and at 0.15625-0.625 μM doxorubicin were <0.
		6.25	0.3125	0.79		4.22				
		12.5	0.625	0.41		2.62				
		25	1.25	0.21	–	1.41	+	0.85	+	
Onco Targets Ther 2021,14,4061		Chidamide (μM)	Lenalidomide (μM)							
	ARP-1	0.5	0.5	1.87	+	1.39	+	0.83	–	
		1	1	2.14	+	1.05	+	0.87	+	
		2	2	2.42	+	1.18	+	1.00	+	
		4	4	2.75	+	1.22	+	1.01	+	
		8	8	3.51	+	2.21	+	1.00	+	
	RPMI-8226	0.5	0.5	1.47	+	0.92	+	0.80	–	
		1	1	2.71	+	1.64	+	1.00	+	
		2	2	4.15	+	2.02	+	1.08	+	
		4	4	7.14	+	3.01	+	1.18	+	
		8	8	11.76	+	7.14	+	1.30	+	
Folia Medica 2021,63, 488		Epirubicin (μM)	Artemisinin (μM)							
	HL-60	1.25	200	2.13	+	2.43	+	1.52	+	
		2.5	400	0.95	+	1.22	+	0.99	+	
		5	800	0.48	–	0.61	–	0.98	+	
	HL-60/Dox	1.25	100	1.69	+	1.67	+	1.57	+	
		2.5	200	1.92	+	1.90	+	5.80	+	
		5	400	3.45	+	3.36	+	1.48	+	

+, beneficial; –, undesirable.

1. Trials released in 2021–2023 were searched in PubMed, using terms “drug combination combination index Chou method”. A trial was included for reevaluations when i) CI-C was listed, and ii) cell-death fractions can be extracted to calculate 1/CI-C and CI-J. Data were extracted in 10/34 papers.
2. A reevaluation with the Chou’s method was performed using the software CalcuSyn (Biosoft, Ferguson, MO, USA).
3. A 1/CI-C of ≥0.91, or a CI-J of ≥0.85 was beneficial; data were compared only when CI-J was available.

REFERENCES

1. Hussain Y, Singh J, Meena A, et al. Escin-sorafenib synergy up-regulates LC3-II and p62 to induce apoptosis in hepatocellular carcinoma cells. Environ Toxicol 2024, 39: 840-856.
2. Gu S, Hou Y, Dovat K, et al. Synergistic effect of HDAC inhibitor chidamide with cladribine on cell cycle arrest and apoptosis by targeting HDAC2/c−Myc/RCC1 axis in acute myeloid leukemia. Exp Hematol Oncol 2023, 12: 23.
3. Li C, Feng Y, Wang W, et al. Targeting glutaminolysis to treat multiple myeloma: An in vitro evaluation of glutaminase inhibitors telaglenastat and epigallocatechin-3-gallate. Anticancer Agents Med Chem 2023, 23: 779-785.
4. Falcão SI, Duarte D, Diallo M, et al. Improvement of the in vitro cytotoxic effect on HT-29 colon cancer cells by combining 5-fluorouacil and fluphenazine with green, red or brown propolis. Molecules 2023, 28: 3393.
5. Duarte D, Falcão SI, El Mehdi I, et al. Honeybee venom synergistically enhances the cytotoxic effect of CNS drugs in HT-29 colon and MCF-7 breast cancer cell lines. Pharmaceutics 2022, 14: 511.
6. Li J, Wu H, Lv S, et al. Enhanced antitumor efficacy by combining afatinib with MDV3100 in castration-resistant prostate cancer. Pharmazie 2022, 77: 59-66.
7. Wang K, Liu W, Xu Q, et al. Tenacissoside G synergistically potentiates inhibitory effects of 5-fluorouracil to human colorectal cancer. Phytomedicine 2021, 86: 153553.
8. McCallister R, Kuszynski D, Cohen MS. Re-evaluation of combinational efficacy and synergy of the Italian protocol in vitro: Are we truly optimizing benefit or permitting unwanted toxicity? Biomedicines 2021, 9: 1190.
9. Jiang D, Zhang K, Zhu Y, et al. Chidamide-induced accumulation of reactive oxygen species increases lenalidomide sensitivity against multiple myeloma cells. Onco Targets Ther 2021, 14: 4061-4075.
10. Zhelyazkova MY, Hristova-Avakumova NG, Momekov GT. Antitumor activity of the combination of artemisinin and epirubicin in human leukemia cells. Folia Med 2021, 63: 488-495.

Table S2. List of 1/CI-C (Chou's method) and CI-J (Jin's method) (simulations at m=2).

Drug A	Drug B	$D_m(A+B)=2$				$D_m(A+B)=4$				$D_m(A+B)=6$				$D_m(A+B)=8$				$D_m(A+B)=10$				$D_m(A+B)=12$				$D_m(A+B)=14$				$D_m(A+B)=16$				$D_m(A+B)=18$				$D_m(A+B)=20$			
$D_m(A)=10$	$D_m(B)=2$	1/CI-C (Chou)		CI-J (Jin)		1/CI-C (Chou)		CI-J (Jin)		1/CI-C (Chou)		CI-J (Jin)		1/CI-C (Chou)		CI-J (Jin)		1/CI-C (Chou)		CI-J (Jin)		1/CI-C (Chou)		CI-J (Jin)		1/CI-C (Chou)		CI-J (Jin)		1/CI-C (Chou)		CI-J (Jin)		1/CI-C (Chou)		CI-J (Jin)		1/CI-C (Chou)		CI-J (Jin)	
1	1	1.67	+	2.40	+	0.83	–	0.96	+	0.56	–	0.48	–	0.42	–	0.28	–	0.33	–	0.18	–	0.28	–	0.13	–	0.24	–	0.10	–	0.21	–	0.07	–	0.19	–	0.06	–	0.17	–	0.05	–
2	2	1.67	+	1.54	+	0.83	–	0.96	+	0.56	–	0.59	–	0.42	–	0.39	–	0.33	–	0.27	–	0.28	–	0.19	–	0.24	–	0.15	–	0.21	–	0.11	–	0.19	–	0.09	–	0.17	–	0.07	–
4	4	1.67	+	1.14	+	0.83	–	0.97	+	0.56	–	0.77	–	0.42	–	0.60	–	0.33	–	0.47	–	0.28	–	0.37	–	0.24	–	0.30	–	0.21	–	0.24	–	0.19	–	0.20	–	0.17	–	0.17	–
8	8	1.67	+	1.02	+	0.83	–	0.98	+	0.56	–	0.91	+	0.42	–	0.83	–	0.33	–	0.75	–	0.28	–	0.66	–	0.24	–	0.59	–	0.21	–	0.52	–	0.19	–	0.46	–	0.17	–	0.40	–
16	16	1.67	+	1.00	+	0.83	–	0.99	+	0.56	–	0.97	+	0.42	–	0.95	+	0.33	–	0.91	+	0.28	–	0.88	+	0.24	–	0.84	–	0.21	–	0.80	–	0.19	–	0.76	–	0.17	–	0.72	–
32	32	1.67	+	1.00	+	0.83	–	1.00	+	0.56	–	0.99	+	0.42	–	0.98	+	0.33	–	0.98	+	0.28	–	0.97	+	0.24	–	0.95	+	0.21	–	0.94	+	0.19	–	0.93	+	0.17	–	0.91	+
$D_m(A)=10$	$D_m(B)=4$																																								
1	1	1.86	+	7.34	+	1.43	+	2.94	+	0.95	+	1.47	+	0.71	–	0.86	+	0.57	–	0.56	–	0.48	–	0.40	–	0.41	–	0.29	–	0.36	–	0.23	–	0.32	–	0.18	–	0.29	–	0.15	–
2	2	1.86	+	3.47	+	1.43	+	2.17	+	0.95	+	1.33	+	0.71	–	0.87	+	0.57	–	0.60	–	0.48	–	0.43	–	0.41	–	0.33	–	0.36	–	0.25	–	0.32	–	0.20	–	0.29	–	0.17	–
4	4	1.86	+	1.65	+	1.43	+	1.41	+	0.95	+	1.12	+	0.71	–	0.88	+	0.57	–	0.69	–	0.48	–	0.54	–	0.41	–	0.43	–	0.36	–	0.35	–	0.32	–	0.29	–	0.29	–	0.24	–
8	8	1.86	+	1.12	+	1.43	+	1.07	+	0.95	+	1.00	+	0.71	–	0.91	+	0.57	–	0.82	–	0.48	–	0.73	–	0.41	–	0.65	–	0.36	–	0.57	–	0.32	–	0.50	–	0.29	–	0.44	–
16	16	1.86	+	1.01	+	1.43	+	1.00	+	0.95	+	0.98	+	0.71	–	0.96	+	0.57	–	0.93	+	0.48	–	0.89	+	0.41	–	0.85	+	0.36	–	0.81	–	0.32	–	0.77	–	0.29	–	0.73	–
32	32	1.86	+	1.00	+	1.43	+	1.00	+	0.95	+	0.99	+	0.71	–	0.99	+	0.57	–	0.98	+	0.48	–	0.97	+	0.41	–	0.96	+	0.36	–	0.94	+	0.32	–	0.93	+	0.29	–	0.91	+
$D_m(A)=10$	$D_m(B)=6$																																								
1	1	3.75	+	13.64	+	1.88	+	5.46	+	1.25	+	2.73	+	0.94	+	1.60	+	0.75	–	1.05	+	0.62	–	0.74	–	0.54	–	0.55	–	0.47	–	0.42	–	0.42	–	0.33	–	0.37	–	0.27	–
2	2	3.75	+	5.94	+	1.88	+	3.71	+	1.25	+	2.29	+	0.94	+	1.49	+	0.75	–	1.02	+	0.62	–	0.74	–	0.54	–	0.56	–	0.47	–	0.44	–	0.42	–	0.35	–	0.37	–	0.29	–
4	4	3.75	+	2.33	+	1.88	+	1.98	+	1.25	+	1.59	+	0.94	+	1.24	+	0.75	–	0.97	+	0.62	–	0.76	–	0.54	–	0.61	–	0.47	–	0.50	–	0.42	–	0.41	–	0.37	–	0.34	–
8	8	3.75	+	1.26	+	1.88	+	1.21	+	1.25	+	1.12	+	0.94	+	1.03	+	0.75	–	0.92	+	0.62	–	0.82	–	0.54	–	0.73	–	0.47	–	0.64	–	0.42	–	0.57	–	0.37	–	0.50	–
16	16	3.75	+	1.03	+	1.88	+	1.02	+	1.25	+	1.00	+	0.94	+	0.97	+	0.75	–	0.94	+	0.62	–	0.91	+	0.54	–	0.87	+	0.47	–	0.83	–	0.42	–	0.79	–	0.37	–	0.74	–
32	32	3.75	+	1.00	+	1.88	+	1.00	+	1.25	+	0.99	+	0.94	+	0.99	+	0.75	–	0.98	+	0.62	–	0.97	+	0.54	–	0.96	+	0.47	–	0.94	+	0.42	–	0.93	+	0.37	–	0.91	+
$D_m(A)=10$	$D_m(B)=8$																																								
1	1	4.44	+	19.89	+	2.22	+	7.96	+	1.48	+	3.98	+	1.11	+	2.34	+	0.89	–	1.53	+	0.74	–	1.08	+	0.63	–	0.80	–	0.56	–	0.61	–	0.49	–	0.49	–	0.44	–	0.39	–
2	2	4.44	+	8.42	+	2.22	+	5.26	+	1.48	+	3.24	+	1.11	+	2.10	+	0.89	–	1.45	+	0.74	–	1.05	+	0.63	–	0.79	–	0.56	–	0.62	–	0.49	–	0.50	–	0.44	–	0.40	–
4	4	4.44	+	3.03	+	2.22	+	2.58	+	1.48	+	2.06	+	1.11	+	1.61	+	0.89	–	1.26	+	0.74	–	0.99	+	0.63	–	0.79	–	0.56	–	0.64	–	0.49	–	0.53	–	0.44	–	0.44	–
8	8	4.44	+	1.42	+	2.22	+	1.35	+	1.48	+	1.26	+	1.11	+	1.15	+	0.89	–	1.03	+	0.74	–	0.92	+	0.63	–	0.81	–	0.56	–	0.72	–	0.49	–	0.63	–	0.44	–	0.56	–
16	16	4.44	+	1.06	+	2.22	+	1.04	+	1.48	+	1.02	+	1.11	+	1.00	+	0.89	–	0.97	+	0.74	–	0.93	+	0.63	–	0.89	+	0.56	–	0.85	+	0.49	–	0.80	–	0.44	–	0.76	–
32	32	4.44	+	1.00	+	2.22	+	1.00	+	1.48	+	1.00	+	1.11	+	0.99	+	0.89	–	0.98	+	0.74	–	0.97	+	0.63	–	0.96	+	0.56	–	0.95	+	0.49	–	0.93	+	0.44	–	0.92	+
$D_m(A)=10$	$D_m(B)=10$																																								
1	1	5.00	+	25.38	+	2.50	+	10.15	+	1.67	+	5.08	+	1.25	+	2.99	+	1.00	+	1.95	+	0.83	–	1.37	+	0.71	–	1.02	+	0.62	–	0.78	–	0.56	–	0.62	–	0.50	–	0.50	–
2	2	5.00	+	10.60	+	2.50	+	6.63	+	1.67	+	4.08	+	1.25	+	2.65	+	1.00	+	1.83	+	0.83	–	1.33	+	0.71	–	1.00	+	0.62	–	0.78	–	0.56	–	0.62	–	0.50	–	0.51	–
4	4	5.00	+	3.66	+	2.50	+	3.11	+	1.67	+	2.49	+	1.25	+	1.95	+	1.00	+	1.52	+	0.83	–	1.20	+	0.71	–	0.96	+	0.62	–	0.78	–	0.56	–	0.64	–	0.50	–	0.54	–
8	8	5.00	+	1.57	+	2.50	+	1.50	+	1.67	+	1.40	+	1.25	+	1.27	+	1.00	+	1.14	+	0.83	–	1.02	+	0.71	–	0.90	+	0.62	–	0.80	–	0.56	–	0.70	–	0.50	–	0.62	–
16	16	5.00	+	1.08	+	2.50	+	1.07	+	1.67	+	1.05	+	1.25	+	1.02	+	1.00	+	0.99	+	0.83	–	0.95	+	0.71	–	0.91	+	0.62	–	0.87	+	0.56	–	0.82	–	0.50	–	0.78	–
32	32	5.00	+	1.01	+	2.50	+	1.00	+	1.67	+	1.00	+	1.25	+	0.99	+	1.00	+	0.98	+	0.83	–	0.97	+	0.71	–	0.96	+	0.62	–	0.95	+	0.56	–	0.93	+	0.50	–	0.92	+
$D_m(A)=10$	$D_m(B)=12$																																								
1	1	5.46	+	29.89	+	2.72	+	11.96	+	1.82	+	5.98	+	1.36	+	3.52	+	1.09	+	2.30	+	0.91	+	1.62	+	0.78	–	1.20	+	0.68	–	0.92	+	0.61	–	0.73	–	0.55	–	0.59	–
2	2	5.46	+	12.41	+	2.72	+	7.76	+	1.82	+	4.77	+	1.36	+	3.10	+	1.09	+	2.14	+	0.91	+	1.55	+	0.78	–	1.17	+	0.68	–	0.91	+	0.61	–	0.73	–	0.55	–	0.60	–
4	4	5.46	+	4.20	+	2.72	+	3.57	+	1.82	+	2.86	+	1.36	+	2.23	+	1.09	+	1.74	+	0.91	+	1.37	+	0.78	–	1.10	+	0.68	–	0.89	+	0.61	–	0.74	–	0.55	–	0.62	–
8	8	5.46	+	1.70	+	2.72	+	1.63	+	1.82	+	1.52	+	1.36	+	1.38	+	1.09	+	1.24	+	0.91	+	1.11	+	0.78	–	0.98	+	0.68	–	0.87	+	0.61	–	0.76	–	0.55	–	0.68	–
16	16	5.46	+	1.11	+	2.72	+	1.10	+	1.82	+	1.07	+	1.36	+	1.05	+	1.09	+	1.01	+	0.91	+	0.98	+	0.78	–	0.93	+	0.68	–	0.89	+	0.61	–	0.85	+	0.55	–	0.80	–
32	32	5.46	+	1.01	+	2.72	+	1.01	+	1.82	+	1.00	+	1.36	+	1.00	+	1.09	+	0.99	+	0.91	+	0.98	+	0.78	–	0.96	+	0.68	–	0.95	+	0.61	–	0.94	+	0.55	–	0.92	+
$D_m(A)=10$	$D_m(B)=14$																																								
1	1	5.85	+	33.50	+	2.92	+	13.40	+	1.95	+	6.70	+	1.46	+	3.94	+	1.17	+	2.58	+	0.97	+	1.81	+	0.83	–	1.34	+	0.73	–	1.03	+	0.65	–	0.82	–	0.58	–	0.66	–
2	2	5.85	+	13.87	+	2.92	+	8.67	+	1.95	+	5.33	+	1.46	+	3.47	+	1.17	+	2.39	+	0.97	+	1.73	+	0.83	–	1.31	+	0.73	–	1.02	+	0.65	–	0.82	–	0.58	–	0.67	–
4	4	5.85	+	4.64	+	2.92	+	3.94	+	1.95	+	3.15	+	1.46	+	2.46	+	1.17	+	1.92	+	0.97	+	1.52	+	0.83	–	1.21	+	0.73	–	0.99	+	0.65	–	0.81	–	0.58	–	0.68	–

8	8	5.85	+	1.82	+	2.92	+	1.74	+	1.95	+	1.62	+	1.46	+	1.48	+	1.17	+	1.33	+	0.97	+	1.18	+	0.83	–	1.05	+	0.73	–	0.93	+	0.65	–	0.82	–	0.58	–	0.72	–
16	16	5.85	+	1.13	+	2.92	+	1.12	+	1.95	+	1.10	+	1.46	+	1.07	+	1.17	+	1.04	+	0.97	+	1.00	+	0.83	–	0.96	+	0.73	–	0.91	+	0.65	–	0.87	+	0.58	–	0.82	–
32	32	5.85	+	1.01	+	2.92	+	1.01	+	1.95	+	1.01	+	1.46	+	1.00	+	1.17	+	0.99	+	0.97	+	0.98	+	0.83	–	0.97	+	0.73	–	0.95	+	0.65	–	0.94	+	0.58	–	0.92	+
$D_m(A)$=10	$D_m(B)$=16																																								
1	1	6.18	+	36.35	+	3.08	+	14.54	+	2.05	+	7.27	+	1.54	+	4.28	+	1.23	+	2.80	+	1.03	+	1.97	+	0.88	–	1.45	+	0.77	–	1.12	+	0.68	–	0.89	+	0.62	–	0.72	–
2	2	6.18	+	15.02	+	3.08	+	9.39	+	2.05	+	5.78	+	1.54	+	3.76	+	1.23	+	2.59	+	1.03	+	1.88	+	0.88	–	1.42	+	0.77	–	1.10	+	0.68	–	0.88	+	0.62	–	0.72	–
4	4	6.18	+	4.99	+	3.08	+	4.24	+	2.05	+	3.39	+	1.54	+	2.65	+	1.23	+	2.07	+	1.03	+	1.63	+	0.88	–	1.30	+	0.77	–	1.06	+	0.68	–	0.87	+	0.62	–	0.73	–
8	8	6.18	+	1.92	+	3.08	+	1.84	+	2.05	+	1.71	+	1.54	+	1.56	+	1.23	+	1.40	+	1.03	+	1.25	+	0.88	–	1.11	+	0.77	–	0.98	+	0.68	–	0.86	+	0.62	–	0.76	–
16	16	6.18	+	1.16	+	3.08	+	1.15	+	2.05	+	1.12	+	1.54	+	1.09	+	1.23	+	1.06	+	1.03	+	1.02	+	0.88	–	0.98	+	0.77	–	0.93	+	0.68	–	0.88	+	0.62	–	0.84	–
32	32	6.18	+	1.02	+	3.08	+	1.01	+	2.05	+	1.01	+	1.54	+	1.00	+	1.23	+	0.99	+	1.03	+	0.98	+	0.88	–	0.97	+	0.77	–	0.96	+	0.68	–	0.94	+	0.62	–	0.93	+
$D_m(A)$=10	$D_m(B)$=18																																								
1	1	6.41	+	38.62	+	3.22	+	15.45	+	2.14	+	7.72	+	1.61	+	4.54	+	1.29	+	2.97	+	1.07	+	2.09	+	0.92	+	1.54	+	0.80	–	1.19	+	0.71	–	0.94	+	0.64	–	0.76	–
2	2	6.41	+	15.94	+	3.22	+	9.96	+	2.14	+	6.13	+	1.61	+	3.99	+	1.29	+	2.75	+	1.07	+	1.99	+	0.92	+	1.50	+	0.80	–	1.17	+	0.71	–	0.94	+	0.64	–	0.77	–
4	4	6.41	+	5.27	+	3.22	+	4.48	+	2.14	+	3.59	+	1.61	+	2.80	+	1.29	+	2.19	+	1.07	+	1.72	+	0.92	+	1.38	+	0.80	–	1.12	+	0.71	–	0.92	+	0.64	–	0.77	–
8	8	6.41	+	2.01	+	3.22	+	1.92	+	2.14	+	1.79	+	1.61	+	1.63	+	1.29	+	1.47	+	1.07	+	1.30	+	0.92	+	1.15	+	0.80	–	1.02	+	0.71	–	0.90	+	0.64	–	0.80	–
16	16	6.41	+	1.18	+	3.22	+	1.17	+	2.14	+	1.15	+	1.61	+	1.12	+	1.29	+	1.08	+	1.07	+	1.04	+	0.92	+	1.00	+	0.80	–	0.95	+	0.71	–	0.90	+	0.64	–	0.85	+
32	32	6.41	+	1.02	+	3.22	+	1.02	+	2.14	+	1.01	+	1.61	+	1.01	+	1.29	+	1.00	+	1.07	+	0.99	+	0.92	+	0.98	+	0.80	–	0.96	+	0.71	–	0.95	+	0.64	–	0.93	+
$D_m(A)$=10	$D_m(B)$=20																																								
1	1	6.67	+	40.42	+	3.33	+	16.17	+	2.22	+	8.08	+	1.67	+	4.76	+	1.33	+	3.11	+	1.11	+	2.18	+	0.95	+	1.62	+	0.83	–	1.24	+	0.74	–	0.99	+	0.67	–	0.80	–
2	2	6.67	+	16.67	+	3.33	+	10.42	+	2.22	+	6.41	+	1.67	+	4.17	+	1.33	+	2.87	+	1.11	+	2.08	+	0.95	+	1.57	+	0.83	–	1.23	+	0.74	–	0.98	+	0.67	–	0.80	–
4	4	6.67	+	5.50	+	3.33	+	4.68	+	2.22	+	3.74	+	1.67	+	2.92	+	1.33	+	2.28	+	1.11	+	1.80	+	0.95	+	1.44	+	0.83	–	1.17	+	0.74	–	0.96	+	0.67	–	0.81	–
8	8	6.67	+	2.08	+	3.33	+	1.98	+	2.22	+	1.85	+	1.67	+	1.69	+	1.33	+	1.52	+	1.11	+	1.35	+	0.95	+	1.19	+	0.83	–	1.05	+	0.74	–	0.93	+	0.67	–	0.82	–
16	16	6.67	+	1.20	+	3.33	+	1.19	+	2.22	+	1.17	+	1.67	+	1.14	+	1.33	+	1.10	+	1.11	+	1.06	+	0.95	+	1.01	+	0.83	–	0.97	+	0.74	–	0.92	+	0.67	–	0.87	+
32	32	6.67	+	1.02	+	3.33	+	1.02	+	2.22	+	1.02	+	1.67	+	1.01	+	1.33	+	1.00	+	1.11	+	0.99	+	0.95	+	0.98	+	0.83	–	0.97	+	0.74	–	0.95	+	0.67	–	0.93	+

Table S3. List of 1/CI-C (Chou's method) and CI-J (Jin's method) (simulations at m=0.5).

**Drug A**	**Drug B**	**D_m(A+B)=2**				**D_m(A+B)=4**				**D_m(A+B)=6**				**D_m(A+B)=8**				**D_m(A+B)=10**				**D_m(A+B)=12**				**D_m(A+B)=14**				**D_m(A+B)=16**				**D_m(A+B)=18**				**D_m(A+B)=20**			
D_m(A)=10	D_m(B)=2	1/CI-C (Chou)		CI-J (Jin)		1/CI-C (Chou)		CI-J (Jin)		1/CI-C (Chou)		CI-J (Jin)		1/CI-C (Chou)		CI-J (Jin)		1/CI-C (Chou)		CI-J (Jin)		1/CI-C (Chou)		CI-J (Jin)		1/CI-C (Chou)		CI-J (Jin)		1/CI-C (Chou)		CI-J (Jin)		1/CI-C (Chou)		CI-J (Jin)		1/CI-C (Chou)		CI-J (Jin)	
1	1	1.67	+	0.90	+	0.83	–	0.75	–	0.56	–	0.66	–	0.42	–	0.60	–	0.33	–	0.56	–	0.28	–	0.52	–	0.24	–	0.49	–	0.21	–	0.47	–	0.19	–	0.45	–	0.17	–	0.43	–
2	2	1.67	+	0.90	+	0.83	–	0.76	–	0.56	–	0.69	–	0.42	–	0.63	–	0.33	–	0.59	–	0.28	–	0.56	–	0.24	–	0.53	–	0.21	–	0.51	–	0.19	–	0.49	–	0.17	–	0.47	–
4	4	1.67	+	0.89	+	0.83	–	0.78	–	0.56	–	0.72	–	0.42	–	0.67	–	0.33	–	0.63	–	0.28	–	0.60	–	0.24	–	0.58	–	0.21	–	0.56	–	0.19	–	0.54	–	0.17	–	0.52	–
8	8	1.67	+	0.90	+	0.83	–	0.81	–	0.56	–	0.75	–	0.42	–	0.71	–	0.33	–	0.68	–	0.28	–	0.65	–	0.24	–	0.63	–	0.21	–	0.61	–	0.19	–	0.59	–	0.17	–	0.57	–
16	16	1.67	+	0.90	+	0.83	–	0.84	–	0.56	–	0.79	–	0.42	–	0.75	–	0.33	–	0.73	–	0.28	–	0.70	–	0.24	–	0.68	–	0.21	–	0.66	–	0.19	–	0.65	–	0.17	–	0.63	–
32	32	1.67	+	0.92	+	0.83	–	0.86	+	0.56	–	0.82	–	0.42	–	0.80	–	0.33	–	0.77	–	0.28	–	0.75	–	0.24	–	0.73	–	0.21	–	0.72	–	0.19	–	0.70	–	0.17	–	0.69	–
D_m(A)=10	D_m(B)=4																																								
1	1	1.86	+	1.01	+	1.43	+	0.84	–	0.95	+	0.74	–	0.71	–	0.68	–	0.57	–	0.63	–	0.48	–	0.59	–	0.41	–	0.56	–	0.36	–	0.53	–	0.32	–	0.51	–	0.29	–	0.49	–
2	2	1.86	+	0.98	+	1.43	+	0.84	–	0.95	+	0.76	–	0.71	–	0.70	–	0.57	–	0.65	–	0.48	–	0.61	–	0.41	–	0.59	–	0.36	–	0.56	–	0.32	–	0.54	–	0.29	–	0.52	–
4	4	1.86	+	0.96	+	1.43	+	0.84	–	0.95	+	0.77	–	0.71	–	0.72	–	0.57	–	0.68	–	0.48	–	0.65	–	0.41	–	0.62	–	0.36	–	0.60	–	0.32	–	0.58	–	0.29	–	0.56	–
8	8	1.86	+	0.95	+	1.43	+	0.85	+	0.95	+	0.79	–	0.71	–	0.75	–	0.57	–	0.71	–	0.48	–	0.69	–	0.41	–	0.66	–	0.36	–	0.64	–	0.32	–	0.62	–	0.29	–	0.60	–
16	16	1.86	+	0.94	+	1.43	+	0.87	+	0.95	+	0.82	–	0.71	–	0.78	–	0.57	–	0.75	–	0.48	–	0.73	–	0.41	–	0.71	–	0.36	–	0.69	–	0.32	–	0.67	–	0.29	–	0.65	–
32	32	1.86	+	0.94	+	1.43	+	0.88	+	0.95	+	0.84	–	0.71	–	0.82	–	0.57	–	0.79	–	0.48	–	0.77	–	0.41	–	0.75	–	0.36	–	0.74	–	0.32	–	0.72	–	0.29	–	0.71	–
D_m(A)=10	D_m(B)=6																																								
1	1	3.75	+	1.09	+	1.88	+	0.90	+	1.25	+	0.79	–	0.94	+	0.72	–	0.75	–	0.67	–	0.62	–	0.63	–	0.54	–	0.60	–	0.47	–	0.57	–	0.42	–	0.54	–	0.37	–	0.52	–
2	2	3.75	+	1.04	+	1.88	+	0.89	+	1.25	+	0.80	–	0.94	+	0.74	–	0.75	–	0.69	–	0.62	–	0.65	–	0.54	–	0.62	–	0.47	–	0.59	–	0.42	–	0.57	–	0.37	–	0.55	–
4	4	3.75	+	1.01	+	1.88	+	0.88	+	1.25	+	0.81	–	0.94	+	0.75	–	0.75	–	0.71	–	0.62	–	0.68	–	0.54	–	0.65	–	0.47	–	0.62	–	0.42	–	0.60	–	0.37	–	0.58	–
8	8	3.75	+	0.98	+	1.88	+	0.88	+	1.25	+	0.82	–	0.94	+	0.78	–	0.75	–	0.74	–	0.62	–	0.71	–	0.54	–	0.68	–	0.47	–	0.66	–	0.42	–	0.64	–	0.37	–	0.63	–
16	16	3.75	+	0.96	+	1.88	+	0.89	+	1.25	+	0.84	–	0.94	+	0.80	–	0.75	–	0.77	–	0.62	–	0.75	–	0.54	–	0.72	–	0.47	–	0.70	–	0.42	–	0.69	–	0.37	–	0.67	–
32	32	3.75	+	0.95	+	1.88	+	0.90	+	1.25	+	0.86	+	0.94	+	0.83	–	0.75	–	0.80	–	0.62	–	0.78	–	0.54	–	0.76	–	0.47	–	0.75	–	0.42	–	0.73	–	0.37	–	0.72	–
D_m(A)=10	D_m(B)=8																																								
1	1	4.44	+	1.14	+	2.22	+	0.94	+	1.48	+	0.83	–	1.11	+	0.76	–	0.89	–	0.70	–	0.74	–	0.66	–	0.63	–	0.63	–	0.56	–	0.60	–	0.49	–	0.57	–	0.44	–	0.55	–
2	2	4.44	+	1.09	+	2.22	+	0.93	+	1.48	+	0.83	–	1.11	+	0.77	–	0.89	–	0.72	–	0.74	–	0.68	–	0.63	–	0.65	–	0.56	–	0.62	–	0.49	–	0.59	–	0.44	–	0.57	–
4	4	4.44	+	1.04	+	2.22	+	0.91	+	1.48	+	0.84	–	1.11	+	0.78	–	0.89	–	0.74	–	0.74	–	0.70	–	0.63	–	0.67	–	0.56	–	0.65	–	0.49	–	0.62	–	0.44	–	0.60	–
8	8	4.44	+	1.00	+	2.22	+	0.91	+	1.48	+	0.84	–	1.11	+	0.80	–	0.89	–	0.76	–	0.74	–	0.73	–	0.63	–	0.70	–	0.56	–	0.68	–	0.49	–	0.66	–	0.44	–	0.64	–
16	16	4.44	+	0.98	+	2.22	+	0.90	+	1.48	+	0.85	+	1.11	+	0.82	–	0.89	–	0.78	–	0.74	–	0.76	–	0.63	–	0.74	–	0.56	–	0.72	–	0.49	–	0.70	–	0.44	–	0.68	–
32	32	4.44	+	0.97	+	2.22	+	0.91	+	1.48	+	0.87	+	1.11	+	0.84	–	0.89	–	0.81	–	0.74	–	0.79	–	0.63	–	0.77	–	0.56	–	0.76	–	0.49	–	0.74	–	0.44	–	0.73	–
D_m(A)=10	D_m(B)=10																																								
1	1	5.00	+	1.18	+	2.50	+	0.98	+	1.67	+	0.87	+	1.25	+	0.79	–	1.00	+	0.73	–	0.83	–	0.69	–	0.71	–	0.65	–	0.62	–	0.62	–	0.56	–	0.59	–	0.50	–	0.57	–
2	2	5.00	+	1.12	+	2.50	+	0.96	+	1.67	+	0.86	+	1.25	+	0.79	–	1.00	+	0.74	–	0.83	–	0.70	–	0.71	–	0.67	–	0.62	–	0.64	–	0.56	–	0.61	–	0.50	–	0.59	–
4	4	5.00	+	1.07	+	2.50	+	0.94	+	1.67	+	0.86	+	1.25	+	0.80	–	1.00	+	0.76	–	0.83	–	0.72	–	0.71	–	0.69	–	0.62	–	0.66	–	0.56	–	0.64	–	0.50	–	0.62	–
8	8	5.00	+	1.02	+	2.50	+	0.92	+	1.67	+	0.86	+	1.25	+	0.81	–	1.00	+	0.77	–	0.83	–	0.74	–	0.71	–	0.72	–	0.62	–	0.69	–	0.56	–	0.67	–	0.50	–	0.65	–
16	16	5.00	+	0.99	+	2.50	+	0.92	+	1.67	+	0.87	+	1.25	+	0.83	–	1.00	+	0.80	–	0.83	–	0.77	–	0.71	–	0.75	–	0.62	–	0.73	–	0.56	–	0.71	–	0.50	–	0.69	–
32	32	5.00	+	0.98	+	2.50	+	0.92	+	1.67	+	0.88	+	1.25	+	0.85	+	1.00	+	0.82	–	0.83	–	0.80	–	0.71	–	0.78	–	0.62	–	0.77	–	0.56	–	0.75	–	0.50	–	0.74	–
D_m(A)=10	D_m(B)=12																																								
1	1	5.46	+	1.22	+	2.72	+	1.01	+	1.82	+	0.89	+	1.36	+	0.81	–	1.09	+	0.75	–	0.91	+	0.71	–	0.78	–	0.67	–	0.68	–	0.64	–	0.61	–	0.61	–	0.55	–	0.59	–
2	2	5.46	+	1.15	+	2.72	+	0.98	+	1.82	+	0.88	+	1.36	+	0.81	–	1.09	+	0.76	–	0.91	+	0.72	–	0.78	–	0.68	–	0.68	–	0.65	–	0.61	–	0.63	–	0.55	–	0.61	–
4	4	5.46	+	1.09	+	2.72	+	0.96	+	1.82	+	0.88	+	1.36	+	0.82	–	1.09	+	0.77	–	0.91	+	0.73	–	0.78	–	0.70	–	0.68	–	0.68	–	0.61	–	0.65	–	0.55	–	0.63	–
8	8	5.46	+	1.04	+	2.72	+	0.94	+	1.82	+	0.87	+	1.36	+	0.83	–	1.09	+	0.79	–	0.91	+	0.76	–	0.78	–	0.73	–	0.68	–	0.70	–	0.61	–	0.68	–	0.55	–	0.67	–
16	16	5.46	+	1.01	+	2.72	+	0.93	+	1.82	+	0.88	+	1.36	+	0.84	–	1.09	+	0.81	–	0.91	+	0.78	–	0.78	–	0.76	–	0.68	–	0.74	–	0.61	–	0.72	–	0.55	–	0.70	–
32	32	5.46	+	0.98	+	2.72	+	0.93	+	1.82	+	0.89	+	1.36	+	0.86	+	1.09	+	0.83	–	0.91	+	0.81	–	0.78	–	0.79	–	0.68	–	0.77	–	0.61	–	0.76	–	0.55	–	0.74	–
D_m(A)=10	D_m(B)=14																																								
1	1	5.85	+	1.25	+	2.92	+	1.03	+	1.95	+	0.91	+	1.46	+	0.83	–	1.17	+	0.77	–	0.97	+	0.72	–	0.83	–	0.68	–	0.73	–	0.65	–	0.65	–	0.62	–	0.58	–	0.60	–
2	2	5.85	+	1.17	+	2.92	+	1.00	+	1.95	+	0.90	+	1.46	+	0.83	–	1.17	+	0.78	–	0.97	+	0.73	–	0.83	–	0.70	–	0.73	–	0.67	–	0.65	–	0.64	–	0.58	–	0.62	–
4	4	5.85	+	1.11	+	2.92	+	0.98	+	1.95	+	0.89	+	1.46	+	0.83	–	1.17	+	0.79	–	0.97	+	0.75	–	0.83	–	0.72	–	0.73	–	0.69	–	0.65	–	0.67	–	0.58	–	0.64	–
8	8	5.85	+	1.06	+	2.92	+	0.95	+	1.95	+	0.89	+	1.46	+	0.84	–	1.17	+	0.80	–	0.97	+	0.77	–	0.83	–	0.74	–	0.73	–	0.71	–	0.65	–	0.69	–	0.58	–	0.68	–
16	16	5.85	+	1.02	+	2.92	+	0.94	+	1.95	+	0.89	+	1.46	+	0.85	+	1.17	+	0.82	–	0.97	+	0.79	–	0.83	–	0.77	–	0.73	–	0.74	–	0.65	–	0.73	–	0.58	–	0.71	–
32	32	5.85	+	0.99	+	2.92	+	0.93	+	1.95	+	0.89	+	1.46	+	0.86	+	1.17	+	0.84	–	0.97	+	0.81	–	0.83	–	0.79	–	0.73	–	0.78	–	0.65	–	0.76	–	0.58	–	0.75	–

$D_m(A)=10$	$D_m(B)=16$																																								
1	1	6.18	+	1.27	+	3.08	+	1.06	+	2.05	+	0.93	+	1.54	+	0.85	+	1.23	+	0.79	–	1.03	+	0.74	–	0.88	–	0.70	–	0.77	–	0.67	–	0.68	–	0.64	–	0.62	–	0.61	–
2	2	6.18	+	1.20	+	3.08	+	1.02	+	2.05	+	0.92	+	1.54	+	0.85	+	1.23	+	0.79	–	1.03	+	0.75	–	0.88	–	0.71	–	0.77	–	0.68	–	0.68	–	0.65	–	0.62	–	0.63	–
4	4	6.18	+	1.13	+	3.08	+	0.99	+	2.05	+	0.91	+	1.54	+	0.85	+	1.23	+	0.80	–	1.03	+	0.76	–	0.88	–	0.73	–	0.77	–	0.70	–	0.68	–	0.68	–	0.62	–	0.65	–
8	8	6.18	+	1.07	+	3.08	+	0.97	+	2.05	+	0.90	+	1.54	+	0.85	+	1.23	+	0.81	–	1.03	+	0.78	–	0.88	–	0.75	–	0.77	–	0.72	–	0.68	–	0.70	–	0.62	–	0.68	–
16	16	6.18	+	1.03	+	3.08	+	0.95	+	2.05	+	0.90	+	1.54	+	0.86	+	1.23	+	0.82	–	1.03	+	0.80	–	0.88	–	0.77	–	0.77	–	0.75	–	0.68	–	0.73	–	0.62	–	0.72	–
32	32	6.18	+	1.00	+	3.08	+	0.94	+	2.05	+	0.90	+	1.54	+	0.87	+	1.23	+	0.84	–	1.03	+	0.82	–	0.88	–	0.80	–	0.77	–	0.78	–	0.68	–	0.77	–	0.62	–	0.75	–
$D_m(A)=10$	$D_m(B)=18$																																								
1	1	6.41	+	1.30	+	3.22	+	1.08	+	2.14	+	0.95	+	1.61	+	0.87	+	1.29	+	0.80	–	1.07	+	0.75	–	0.92	+	0.71	–	0.80	–	0.68	–	0.71	–	0.63	–	0.64	–	0.62	–
2	2	6.41	+	1.22	+	3.22	+	1.04	+	2.14	+	0.93	+	1.61	+	0.86	+	1.29	+	0.80	–	1.07	+	0.76	–	0.92	+	0.72	–	0.80	–	0.69	–	0.71	–	0.67	–	0.64	–	0.64	–
4	4	6.41	+	1.14	+	3.22	+	1.00	+	2.14	+	0.92	+	1.61	+	0.86	+	1.29	+	0.81	–	1.07	+	0.77	–	0.92	+	0.74	–	0.80	–	0.71	–	0.71	–	0.69	–	0.64	–	0.66	–
8	8	6.41	+	1.08	+	3.22	+	0.98	+	2.14	+	0.91	+	1.61	+	0.86	+	1.29	+	0.82	–	1.07	+	0.78	–	0.92	+	0.76	–	0.80	–	0.73	–	0.71	–	0.71	–	0.64	–	0.69	–
16	16	6.41	+	1.04	+	3.22	+	0.96	+	2.14	+	0.90	+	1.61	+	0.86	+	1.29	+	0.83	–	1.07	+	0.80	–	0.92	+	0.78	–	0.80	–	0.76	–	0.71	–	0.74	–	0.64	–	0.72	–
32	32	6.41	+	1.00	+	3.22	+	0.95	+	2.14	+	0.90	+	1.61	+	0.87	+	1.29	+	0.85	+	1.07	+	0.82	–	0.92	+	0.81	–	0.80	–	0.79	–	0.71	–	0.77	–	0.64	–	0.76	–
$D_m(A)=10$	$D_m(B)=20$																																								
1	1	6.67	+	1.32	+	3.33	+	1.09	+	2.22	+	0.97	+	1.67	+	0.88	+	1.33	+	0.82	–	1.11	+	0.76	–	0.95	+	0.72	–	0.83	–	0.69	–	0.74	–	0.66	–	0.67	–	0.63	–
2	2	6.67	+	1.23	+	3.33	+	1.05	+	2.22	+	0.95	+	1.67	+	0.87	+	1.33	+	0.82	–	1.11	+	0.77	–	0.95	+	0.73	–	0.83	–	0.70	–	0.74	–	0.67	–	0.67	–	0.65	–
4	4	6.67	+	1.16	+	3.33	+	1.02	+	2.22	+	0.93	+	1.67	+	0.87	+	1.33	+	0.82	–	1.11	+	0.78	–	0.95	+	0.75	–	0.83	–	0.72	–	0.74	–	0.69	–	0.67	–	0.67	–
8	8	6.67	+	1.09	+	3.33	+	0.99	+	2.22	+	0.92	+	1.67	+	0.87	+	1.33	+	0.83	–	1.11	+	0.79	–	0.95	+	0.76	–	0.83	–	0.74	–	0.74	–	0.72	–	0.67	–	0.70	–
16	16	6.67	+	1.04	+	3.33	+	0.96	+	2.22	+	0.91	+	1.67	+	0.87	+	1.33	+	0.84	–	1.11	+	0.81	–	0.95	+	0.78	–	0.83	–	0.76	–	0.74	–	0.75	–	0.67	–	0.73	–
32	32	6.67	+	1.01	+	3.33	+	0.95	+	2.22	+	0.91	+	1.67	+	0.88	+	1.33	+	0.85	+	1.11	+	0.83	–	0.95	+	0.81	–	0.83	–	0.79	–	0.74	–	0.78	–	0.67	–	0.76	–

Table S4. List of 1/CI-C (Chou's method) and CI-J (Jin's method) (simulations at m=1).

Drug A	Drug B	$D_m(A+B)=2$				$D_m(A+B)=4$				$D_m(A+B)=6$				$D_m(A+B)=8$				$D_m(A+B)=10$				$D_m(A+B)=12$				$D_m(A+B)=14$				$D_m(A+B)=16$				$D_m(A+B)=18$				$D_m(A+B)=20$			
$D_m(A)=10$	$D_m(B)=2$	1/CI-C (Chou)		CI-J (Jin)		1/CI-C (Chou)		CI-J (Jin)		1/CI-C (Chou)		CI-J (Jin)		1/CI-C (Chou)		CI-J (Jin)		1/CI-C (Chou)		CI-J (Jin)		1/CI-C (Chou)		CI-J (Jin)		1/CI-C (Chou)		CI-J (Jin)		1/CI-C (Chou)		CI-J (Jin)		1/CI-C (Chou)		CI-J (Jin)		1/CI-C (Chou)		CI-J (Jin)	
1	1	1.67	+	1.27	+	0.83	–	0.85	+	0.56	–	0.63	–	0.42	–	0.51	–	0.33	–	0.42	–	0.28	–	0.36	–	0.24	–	0.32	–	0.21	–	0.28	–	0.19	–	0.25	–	0.17	–	0.23	–
2	2	1.67	+	1.14	+	0.83	–	0.86	+	0.56	–	0.69	–	0.42	–	0.57	–	0.33	–	0.49	–	0.28	–	0.43	–	0.24	–	0.38	–	0.21	–	0.34	–	0.19	–	0.31	–	0.17	–	0.29	–
4	4	1.67	+	1.05	+	0.83	–	0.88	+	0.56	–	0.75	–	0.42	–	0.66	–	0.33	–	0.58	–	0.28	–	0.53	–	0.24	–	0.48	–	0.21	–	0.44	–	0.19	–	0.40	–	0.17	–	0.38	–
8	8	1.67	+	1.00	+	0.83	–	0.90	+	0.56	–	0.82	–	0.42	–	0.75	–	0.33	–	0.69	–	0.28	–	0.64	–	0.24	–	0.60	–	0.21	–	0.56	–	0.19	–	0.53	–	0.17	–	0.50	–
16	16	1.67	+	0.98	+	0.83	–	0.93	+	0.56	–	0.88	+	0.42	–	0.84	–	0.33	–	0.80	–	0.28	–	0.76	–	0.24	–	0.73	–	0.21	–	0.70	–	0.19	–	0.67	–	0.17	–	0.64	–
32	32	1.67	+	0.98	+	0.83	–	0.95	+	0.56	–	0.93	+	0.42	–	0.90	+	0.33	–	0.88	+	0.28	–	0.85	+	0.24	–	0.83	–	0.21	–	0.81	–	0.19	–	0.79	–	0.17	–	0.77	–
$D_m(A)=10$	$D_m(B)=4$																																								
1	1	1.86	+	1.83	+	1.43	+	1.22	+	0.95	+	0.92	+	0.71	–	0.73	–	0.57	–	0.61	–	0.48	–	0.52	–	0.41	–	0.46	–	0.36	–	0.41	–	0.32	–	0.37	–	0.29	–	0.33	–
2	2	1.86	+	1.50	+	1.43	+	1.13	+	0.95	+	0.90	+	0.71	–	0.75	–	0.57	–	0.64	–	0.48	–	0.56	–	0.41	–	0.50	–	0.36	–	0.45	–	0.32	–	0.41	–	0.29	–	0.38	–
4	4	1.86	+	1.24	+	1.43	+	1.04	+	0.95	+	0.89	+	0.71	–	0.78	–	0.57	–	0.69	–	0.48	–	0.62	–	0.41	–	0.57	–	0.36	–	0.52	–	0.32	–	0.48	–	0.29	–	0.44	–
8	8	1.86	+	1.09	+	1.43	+	0.98	+	0.95	+	0.89	+	0.71	–	0.82	–	0.57	–	0.76	–	0.48	–	0.70	–	0.41	–	0.65	–	0.36	–	0.61	–	0.32	–	0.58	–	0.29	–	0.55	–
16	16	1.86	+	1.02	+	1.43	+	0.96	+	0.95	+	0.91	+	0.71	–	0.87	+	0.57	–	0.83	–	0.48	–	0.79	–	0.41	–	0.75	–	0.36	–	0.72	–	0.32	–	0.69	–	0.29	–	0.67	–
32	32	1.86	+	1.00	+	1.43	+	0.97	+	0.95	+	0.94	+	0.71	–	0.91	+	0.57	–	0.89	+	0.48	–	0.86	+	0.41	–	0.84	–	0.36	–	0.82	–	0.32	–	0.80	–	0.29	–	0.78	–
$D_m(A)=10$	$D_m(B)=6$																																								
1	1	3.75	+	2.26	+	1.88	+	1.51	+	1.25	+	1.13	+	0.94	+	0.91	+	0.75	–	0.75	–	0.62	–	0.65	–	0.54	–	0.57	–	0.47	–	0.50	–	0.42	–	0.45	–	0.37	–	0.41	–
2	2	3.75	+	1.78	+	1.88	+	1.33	+	1.25	+	1.07	+	0.94	+	0.89	+	0.75	–	0.76	–	0.62	–	0.67	–	0.54	–	0.59	–	0.47	–	0.53	–	0.42	–	0.48	–	0.37	–	0.44	–
4	4	3.75	+	1.40	+	1.88	+	1.17	+	1.25	+	1.00	+	0.94	+	0.88	+	0.75	–	0.78	–	0.62	–	0.70	–	0.54	–	0.64	–	0.47	–	0.58	–	0.42	–	0.54	–	0.37	–	0.50	–
8	8	3.75	+	1.17	+	1.88	+	1.05	+	1.25	+	0.95	+	0.94	+	0.88	+	0.75	–	0.81	–	0.62	–	0.75	–	0.54	–	0.70	–	0.47	–	0.66	–	0.42	–	0.62	–	0.37	–	0.58	–
16	16	3.75	+	1.05	+	1.88	+	0.99	+	1.25	+	0.94	+	0.94	+	0.89	+	0.75	–	0.85	+	0.62	–	0.81	–	0.54	–	0.78	–	0.47	–	0.74	–	0.42	–	0.72	–	0.37	–	0.69	–
32	32	3.75	+	1.01	+	1.88	+	0.98	+	1.25	+	0.95	+	0.94	+	0.92	+	0.75	–	0.90	+	0.62	–	0.88	+	0.54	–	0.85	+	0.47	–	0.83	–	0.42	–	0.81	–	0.37	–	0.79	–
$D_m(A)=10$	$D_m(B)=8$																																								
1	1	4.44	+	2.61	+	2.22	+	1.74	+	1.48	+	1.30	+	1.11	+	1.04	+	0.89	–	0.87	+	0.74	–	0.74	–	0.63	–	0.65	–	0.56	–	0.58	–	0.49	–	0.52	–	0.44	–	0.47	–
2	2	4.44	+	2.00	+	2.22	+	1.50	+	1.48	+	1.20	+	1.11	+	1.00	+	0.89	–	0.86	+	0.74	–	0.75	–	0.63	–	0.67	–	0.56	–	0.60	–	0.49	–	0.55	–	0.44	–	0.50	–
4	4	4.44	+	1.53	+	2.22	+	1.27	+	1.48	+	1.09	+	1.11	+	0.95	+	0.89	–	0.85	+	0.74	–	0.76	–	0.63	–	0.69	–	0.56	–	0.64	–	0.49	–	0.59	–	0.44	–	0.55	–
8	8	4.44	+	1.23	+	2.22	+	1.11	+	1.48	+	1.01	+	1.11	+	0.92	+	0.89	–	0.85	+	0.74	–	0.79	–	0.63	–	0.74	–	0.56	–	0.69	–	0.49	–	0.65	–	0.44	–	0.62	–
16	16	4.44	+	1.08	+	2.22	+	1.02	+	1.48	+	0.97	+	1.11	+	0.92	+	0.89	–	0.87	+	0.74	–	0.83	–	0.63	–	0.80	–	0.56	–	0.76	–	0.49	–	0.73	–	0.44	–	0.71	–
32	32	4.44	+	1.02	+	2.22	+	0.99	+	1.48	+	0.96	+	1.11	+	0.93	+	0.89	–	0.91	+	0.74	–	0.88	+	0.63	–	0.86	+	0.56	–	0.84	–	0.49	–	0.82	–	0.44	–	0.80	–
$D_m(A)=10$	$D_m(B)=10$																																								
1	1	5.00	+	2.88	+	2.50	+	1.92	+	1.67	+	1.44	+	1.25	+	1.15	+	1.00	+	0.96	+	0.83	–	0.82	–	0.71	–	0.72	–	0.62	–	0.64	–	0.56	–	0.58	–	0.50	–	0.52	–
2	2	5.00	+	2.18	+	2.50	+	1.64	+	1.67	+	1.31	+	1.25	+	1.09	+	1.00	+	0.94	+	0.83	–	0.82	–	0.71	–	0.73	–	0.62	–	0.65	–	0.56	–	0.60	–	0.50	–	0.55	–
4	4	5.00	+	1.63	+	2.50	+	1.36	+	1.67	+	1.17	+	1.25	+	1.02	+	1.00	+	0.91	+	0.83	–	0.82	–	0.71	–	0.74	–	0.62	–	0.68	–	0.56	–	0.63	–	0.50	–	0.58	–
8	8	5.00	+	1.29	+	2.50	+	1.16	+	1.67	+	1.05	+	1.25	+	0.96	+	1.00	+	0.89	+	0.83	–	0.83	–	0.71	–	0.77	–	0.62	–	0.72	–	0.56	–	0.68	–	0.50	–	0.64	–
16	16	5.00	+	1.10	+	2.50	+	1.04	+	1.67	+	0.99	+	1.25	+	0.94	+	1.00	+	0.89	+	0.83	–	0.85	+	0.71	–	0.82	–	0.62	–	0.78	–	0.56	–	0.75	–	0.50	–	0.72	–
32	32	5.00	+	1.03	+	2.50	+	1.00	+	1.67	+	0.97	+	1.25	+	0.94	+	1.00	+	0.92	+	0.83	–	0.89	+	0.71	–	0.87	+	0.62	–	0.85	+	0.56	–	0.83	–	0.50	–	0.81	–
$D_m(A)=10$	$D_m(B)=12$																																								
1	1	5.46	+	3.11	+	2.72	+	2.07	+	1.82	+	1.55	+	1.36	+	1.24	+	1.09	+	1.04	+	0.91	+	0.89	+	0.78	–	0.78	–	0.68	–	0.69	–	0.61	–	0.62	–	0.55	–	0.57	–
2	2	5.46	+	2.33	+	2.72	+	1.75	+	1.82	+	1.40	+	1.36	+	1.17	+	1.09	+	1.00	+	0.91	+	0.88	+	0.78	–	0.78	–	0.68	–	0.70	–	0.61	–	0.64	–	0.55	–	0.58	–
4	4	5.46	+	1.72	+	2.72	+	1.44	+	1.82	+	1.23	+	1.36	+	1.08	+	1.09	+	0.96	+	0.91	+	0.86	+	0.78	–	0.78	–	0.68	–	0.72	–	0.61	–	0.66	–	0.55	–	0.62	–
8	8	5.46	+	1.33	+	2.72	+	1.20	+	1.82	+	1.09	+	1.36	+	1.00	+	1.09	+	0.92	+	0.91	+	0.86	+	0.78	–	0.80	–	0.68	–	0.75	–	0.61	–	0.71	–	0.55	–	0.67	–
16	16	5.46	+	1.13	+	2.72	+	1.06	+	1.82	+	1.01	+	1.36	+	0.96	+	1.09	+	0.91	+	0.91	+	0.87	+	0.78	–	0.83	–	0.68	–	0.80	–	0.61	–	0.77	–	0.55	–	0.74	–
32	32	5.46	+	1.04	+	2.72	+	1.01	+	1.82	+	0.98	+	1.36	+	0.95	+	1.09	+	0.92	+	0.91	+	0.90	+	0.78	–	0.88	+	0.68	–	0.86	+	0.61	–	0.83	–	0.55	–	0.81	–
$D_m(A)=10$	$D_m(B)=14$																																								
1	1	5.85	+	3.30	+	2.92	+	2.20	+	1.95	+	1.65	+	1.46	+	1.32	+	1.17	+	1.10	+	0.97	+	0.94	+	0.83	–	0.83	–	0.73	–	0.73	–	0.65	–	0.66	–	0.58	–	0.60	–
2	2	5.85	+	2.46	+	2.92	+	1.85	+	1.95	+	1.48	+	1.46	+	1.23	+	1.17	+	1.05	+	0.97	+	0.92	+	0.83	–	0.82	–	0.73	–	0.74	–	0.65	–	0.67	–	0.58	–	0.62	–
4	4	5.85	+	1.80	+	2.92	+	1.50	+	1.95	+	1.29	+	1.46	+	1.13	+	1.17	+	1.00	+	0.97	+	0.90	+	0.83	–	0.82	–	0.73	–	0.75	–	0.65	–	0.69	–	0.58	–	0.64	–
8	8	5.85	+	1.38	+	2.92	+	1.24	+	1.95	+	1.13	+	1.46	+	1.03	+	1.17	+	0.95	+	0.97	+	0.88	+	0.83	–	0.83	–	0.73	–	0.77	–	0.65	–	0.73	–	0.58	–	0.69	–
16	16	5.85	+	1.15	+	2.92	+	1.08	+	1.95	+	1.03	+	1.46	+	0.98	+	1.17	+	0.93	+	0.97	+	0.89	+	0.83	–	0.85	+	0.73	–	0.81	–	0.65	–	0.78	–	0.58	–	0.75	–
32	32	5.85	+	1.05	+	2.92	+	1.01	+	1.95	+	0.99	+	1.46	+	0.96	+	1.17	+	0.93	+	0.97	+	0.91	+	0.83	–	0.88	+	0.73	–	0.86	+	0.65	–	0.84	–	0.58	–	0.82	–

D_m(A)=10	D_m(B)=16																																								
1	1	6.18	+	3.46	+	3.08	+	2.31	+	2.05	+	1.73	+	1.54	+	1.39	+	1.23	+	1.15	+	1.03	+	0.99	+	0.88	–	0.87	+	0.77	–	0.77	–	0.68	–	0.69	–	0.62	–	0.63	–
2	2	6.18	+	2.57	+	3.08	+	1.93	+	2.05	+	1.54	+	1.54	+	1.29	+	1.23	+	1.10	+	1.03	+	0.96	+	0.88	–	0.86	+	0.77	–	0.77	–	0.68	–	0.70	–	0.62	–	0.64	–
4	4	6.18	+	1.87	+	3.08	+	1.56	+	2.05	+	1.33	+	1.54	+	1.17	+	1.23	+	1.04	+	1.03	+	0.93	+	0.88	–	0.85	+	0.77	–	0.78	–	0.68	–	0.72	–	0.62	–	0.67	–
8	8	6.18	+	1.41	+	3.08	+	1.27	+	2.05	+	1.16	+	1.54	+	1.06	+	1.23	+	0.98	+	1.03	+	0.91	+	0.88	–	0.85	+	0.77	–	0.79	–	0.68	–	0.75	–	0.62	–	0.71	–
16	16	6.18	+	1.17	+	3.08	+	1.10	+	2.05	+	1.04	+	1.54	+	0.99	+	1.23	+	0.94	+	1.03	+	0.90	+	0.88	–	0.86	+	0.77	–	0.83	–	0.68	–	0.79	–	0.62	–	0.76	–
32	32	6.18	+	1.05	+	3.08	+	1.02	+	2.05	+	0.99	+	1.54	+	0.97	+	1.23	+	0.94	+	1.03	+	0.91	+	0.88	–	0.89	+	0.77	–	0.87	+	0.68	–	0.85	+	0.62	–	0.83	–
D_m(A)=10	D_m(B)=18																																								
1	1	6.41	+	3.60	+	3.22	+	2.40	+	2.14	+	1.80	+	1.61	+	1.44	+	1.29	+	1.20	+	1.07	+	1.03	+	0.92	+	0.90	+	0.80	–	0.80	–	0.71	–	0.72	–	0.64	–	0.66	–
2	2	6.41	+	2.67	+	3.22	+	2.00	+	2.14	+	1.60	+	1.61	+	1.33	+	1.29	+	1.14	+	1.07	+	1.00	+	0.92	+	0.89	+	0.80	–	0.80	–	0.71	–	0.73	–	0.64	–	0.67	–
4	4	6.41	+	1.93	+	3.22	+	1.60	+	2.14	+	1.38	+	1.61	+	1.20	+	1.29	+	1.07	+	1.07	+	0.96	+	0.92	+	0.88	+	0.80	–	0.80	–	0.71	–	0.74	–	0.64	–	0.69	–
8	8	6.41	+	1.44	+	3.22	+	1.30	+	2.14	+	1.18	+	1.61	+	1.08	+	1.29	+	1.00	+	1.07	+	0.93	+	0.92	+	0.87	+	0.80	–	0.81	–	0.71	–	0.76	–	0.64	–	0.72	–
16	16	6.41	+	1.18	+	3.22	+	1.12	+	2.14	+	1.06	+	1.61	+	1.00	+	1.29	+	0.96	+	1.07	+	0.91	+	0.92	+	0.87	+	0.80	–	0.84	–	0.71	–	0.80	–	0.64	–	0.77	–
32	32	6.41	+	1.06	+	3.22	+	1.03	+	2.14	+	1.00	+	1.61	+	0.97	+	1.29	+	0.95	+	1.07	+	0.92	+	0.92	+	0.90	+	0.80	–	0.88	+	0.71	–	0.85	+	0.64	–	0.83	–
D_m(A)=10	D_m(B)=20																																								
1	1	6.67	+	3.73	+	3.33	+	2.48	+	2.22	+	1.86	+	1.67	+	1.49	+	1.33	+	1.24	+	1.11	+	1.06	+	0.95	+	0.93	+	0.83	–	0.83	–	0.74	–	0.75	–	0.67	–	0.68	–
2	2	6.67	+	2.75	+	3.33	+	2.06	+	2.22	+	1.65	+	1.67	+	1.38	+	1.33	+	1.18	+	1.11	+	1.03	+	0.95	+	0.92	+	0.83	–	0.83	–	0.74	–	0.75	–	0.67	–	0.69	–
4	4	6.67	+	1.98	+	3.33	+	1.65	+	2.22	+	1.41	+	1.67	+	1.24	+	1.33	+	1.10	+	1.11	+	0.99	+	0.95	+	0.90	+	0.83	–	0.82	–	0.74	–	0.76	–	0.67	–	0.71	–
8	8	6.67	+	1.47	+	3.33	+	1.33	+	2.22	+	1.21	+	1.67	+	1.11	+	1.33	+	1.02	+	1.11	+	0.95	+	0.95	+	0.88	+	0.83	–	0.83	–	0.74	–	0.78	–	0.67	–	0.74	–
16	16	6.67	+	1.20	+	3.33	+	1.13	+	2.22	+	1.07	+	1.67	+	1.02	+	1.33	+	0.97	+	1.11	+	0.92	+	0.95	+	0.88	+	0.83	–	0.85	+	0.74	–	0.81	–	0.67	–	0.78	–
32	32	6.67	+	1.07	+	3.33	+	1.04	+	2.22	+	1.01	+	1.67	+	0.98	+	1.33	+	0.95	+	1.11	+	0.93	+	0.95	+	0.90	+	0.83	–	0.88	+	0.74	–	0.86	+	0.67	–	0.84	–

Table S5. A comparison of the Chou's and Jin's methods.

	**Chou's Method**	**Jin's Method**
Combination index (CI)	Using actual ($D_{A\|A+B}$ and $D_{B\|A+B}$ for the combination) and theoretical (D_A and D_B for a drug alone) doses.<br>CI = ($D_{A\|A+B}/D_A$)+($D_{B\|A+B}/D_B$)	Using effects observed (E_A, E_B and E_{A+B}).<br>CI = $E_{A+B}/(E_A+E_B-E_A \times E_B)$
Interpretation of CI	<0.90, synergism (1/CI >1.11);<br>0.90−1.10, addition (1/CI = 0.91−1.11);<br>>1.10, antagonism (1/CI <0.91).	>1.15, synergism;<br>0.85−1.15, addition;<br><0.85, antagonism.
Dose setting	≥3 doses to acquire the dose-effect feature of a drug alone (≥5 doses can outline the dose-effect feature accurately; that 0.5 is within the effect is optimum).<br>Doses of A or B in the combination can differ from those in describing the dose-effect feature of A or B alone.<br>Using a constant dose ratio is the most effective.	Doses of A and B in E_{A+B} should be equal to those in E_A and E_B, respectively.
Data point	≥2 rational data points for a drug alone to outline the dose-effect feature (≥5 data points is optimum).<br>No minimal requirement of data points for the combination.	≥1 data batch (1 batch comprises 3 data points):<br>3+2n data points when fixing the dose of A or B (n = 0, 1…);<br>3+3n data points when not fixing the dose of A or B.
Accuracy	Depending on D_A and D_B.<br>Misjudging the interaction when i) the dose-effect feature of A or B alone is poorly understood, ii) both D_m(A) and D_m(B) are extrapolated, iii) the gap between D_m(A+B) and D_m(A) (or D_m(B)) is small.	Depending on the expected effect ($E_A+E_B-E_A \times E_B$).<br>Not beneficial to detecting antagonism when E_A or E_B is <0.05.<br>Not beneficial to detecting synergism when E_A or E_B is >0.8.
Application	Being easy for in vitro therapy.<br>Limited for in vivo therapy when the dose-effect feature of a drug alone is impossible.	Being easy for in vitro and in vivo therapies.

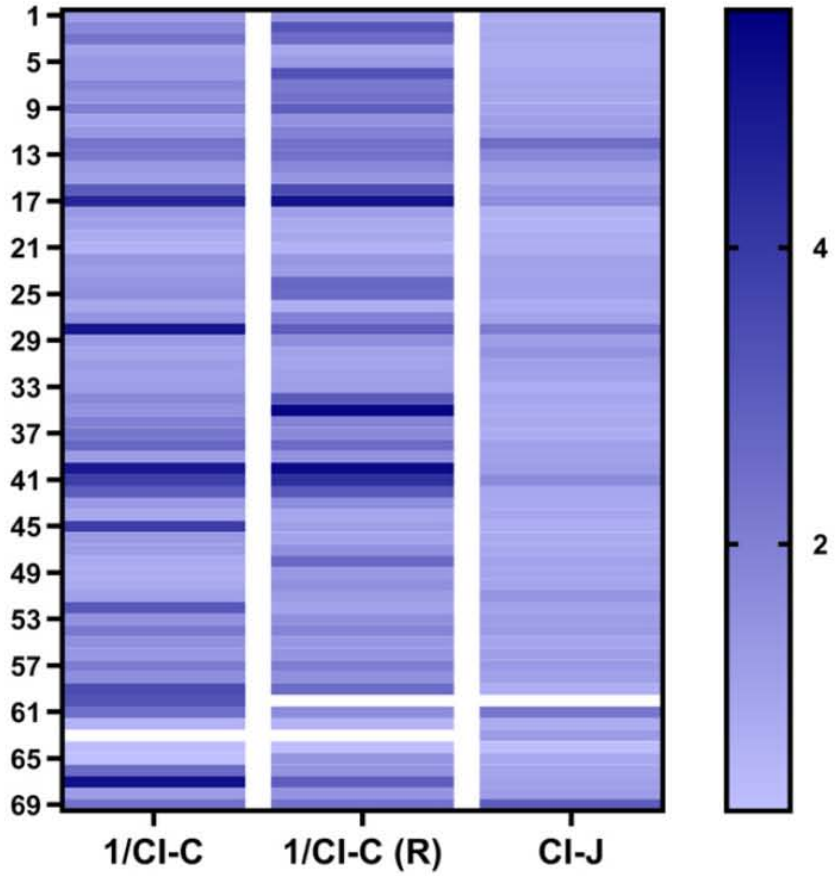

Figure S1. 1/CI-C and CI-J on released data: the mean in each trial was listed, and 1/CI-C (R) was the recalculated value (data were from Table S1). CI-C: combination index from the Chou's method; CI-J: combination index from the Jin's method.

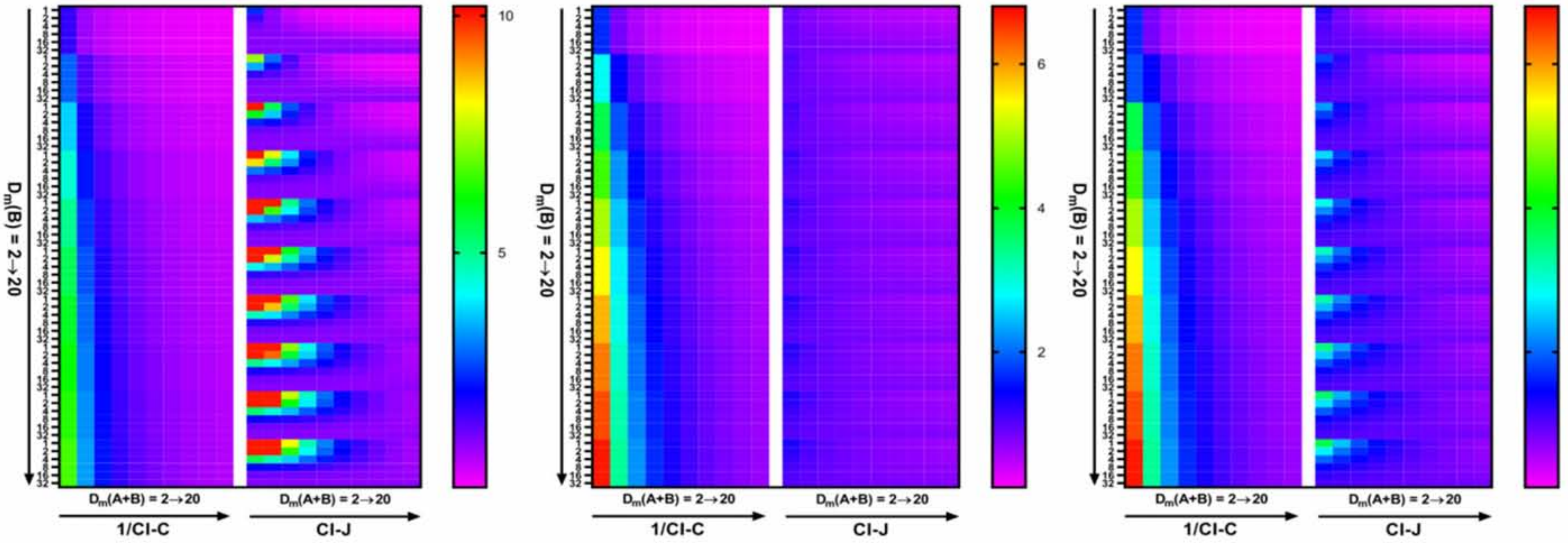

Figure S2. 1/CI-C and CI-J on simulated data at m=2 (*left*), 0.5 (*middle*) and 1 (*right*) (data were from Table S2–S4). CI-C: combination index from the Chou's method; CI-J: combination index from the Jin's method.
